# Actuator and Sensor Fault Detection and Isolation for T-S Fuzzy Systems Based on Zonotopic Set-Membership

**DOI:** 10.3390/s26175365

**Published:** 2026-08-25

**Authors:** Cuicui Li, Fanglai Zhu, Xufeng Ling

**Affiliations:** 1 College of Electronics and Information Engineering, Tongji University, Shanghai 201804, China; 2380122@tongji.edu.cn; 2 School of Intelligent Manufacturing, Liuzhou Railway Vocational Technical College, Liuzhou 545006, China; 3 School of Artificial Intelligence, Shanghai Normal University Tianhua College, Shanghai 201815, China; lxf1131@sthu.edu.cn

**Keywords:** interval state estimation, fault detection, fault isolation, T-S fuzzy systems, zonotope, set-membership

## Abstract

This paper investigates the fault-detection and isolation problems for T-S fuzzy systems with actuator faults and sensor faults. To begin with, the zonotopic set-memberships of both the state and output are set up. After this, by taking the intersection of these two zonotopic set-memberships as the state-estimation zonotopic set-membership, an interval state estimation method is developed. In order to obtain an optimization-interval estimation, a parameterized correction matrix is introduced into the zonotopic set-membership construction. Aiming at some optimization goal, the computation of the parameterized correction matrix is given by LMIs. Moreover, fault detections for both actuator and sensor faults are accomplished, and fault isolation between actuator and sensor faults is also fulfilled by constructing proper residuals using the optimization-state interval estimation. Finally, a simulation example is given to verify the effectiveness of the proposed method.

## 1. Introduction

The core applications of the Takagi–Sugeno (T-S) fuzzy models are used to handle complex nonlinear systems. Through the combination of fuzzy rules and local linear models, the modeling of some complicated nonlinear system is realized by the T-S fuzzy model. Since T-S fuzzy systems can characterize a large class of nonlinear systems, they are widely used in many fields, such as industrial process control, prediction and fault detection, and intelligent systems [1,2,3,4,5,6,7,8,9]. For example, in order to address the time-varying uncertainties and nonlinearities of the rolling model, Reference [1] reconstructs the model into a T-S fuzzy model with uncertainties, then designs a Model Predictive Control (MPC) strategy based on a nonlinear extended state observer for fulfilling ship-fin stabilizable control. Reference [2] designs an observer-based adaptive control for a class of nonlinear stochastic switched systems by using the sliding-mode technique via a T-S fuzzy model. In [3], an interval-type second-order fuzzy disturbance observer is proposed, and a trajectory tracking control scheme is developed, exhibiting strong robustness against external disturbances. Reference [4] constructs a control framework and establishes a T-S fuzzy model for nonlinear cyber-physical systems (CPSs) with faults and attacks and verifies the effectiveness of the proposed control method by using the classical quadruple-tank model.

Fault detection and fault isolation (FDI) based on the T-S fuzzy model represent a popular research direction at present. The FDI methods of T-S fuzzy models include the observer-based method, data-driven method, and optimization algorithm-based method. In the observer-based fault detection (FD) method, a disturbance-resistant but fault-sensitive state observer is designed and used as a fault detector such that FD can be realized by constructing proper residuals using the outputs of both the original system and the observer [10,11,12,13,14,15]. Reference [10] discusses the issue of estimating actuator and sensor faults for an uncertain fractional-order nonlinear system that is characterized by a T-S fuzzy model, and a T-S fuzzy fractional sliding-mode observer and a T-S fuzzy adaptive observer are constructed to deal with the actuator and sensor faults, respectively. For a class of T-S fuzzy singular systems, Reference [11] investigates the problem of FD, where a $H_{-}/H_{\infty }$ residual is generated for fault detection based on a sliding-mode observer. Reference [14] presents a fault detection and isolation scheme for discrete-time Lipschitz nonlinear T-S fuzzy systems based on $H_{\infty }$ observer and zonotopic set-membership estimation. It utilizes zonotope order reduction and two kinds of augmented observers to achieve residual-based fault detection and decouple actuator and sensor faults for isolation.

As an important branch of set-membership estimation, Zonotopic Set-Membership (ZSM) is a state set-valued estimation method designed specially for systems with bounded unknown noise and uncertainties by taking zonotopes as the set representations. Its core lies in constructing a zonotope that contains the true state of the system through a recursive prediction-correction process. Set-membership estimation only requires the knowledge of the boundary ranges of uncertainties to effectively handle system uncertainty through set operations. Therefore, the methods are particularly suitable for industrial scenarios where accurate modeling is difficult or when systems suffer from external disturbances. Set-membership estimation methods can balance estimation accuracy and computational complexity are therefore widely applied in fields like state estimation and parameter identification [16,17,18,19,20,21,22,23]. Reference [16] proposes an innovative nonlinear zonotopic set-membership estimation (SME) framework that adopts the Koopman operator and deep neural networks (DNNs) to map the primitive nonlinear system to a linear system in higher-dimensional space. Reference [18] solves the stability problems for classical linear time-invariant systems, where a new concept named the observation-information tower is developed to describe the effectsof the measurements on the SME without relying on the initial condition. Studies have also improved the adaptability of zonotopes to complex uncertainties by combining machine learning methods, further reducing estimation conservatism [22,23]. In order to reduce the conservatism in SME, optimization techniques have been employed to SME analysis [24,25,26,27,28]. For T-S fuzzy systems, obtaining a state estimation based on traditional observer techniques is not a trivial task [29,30,31]. Reference [29] designs a Kalman-type observer for T-S fuzzy bilinear systems and derives sufficient conditions to guarantee the exponential convergence of observation errors. It can handle bounded uncertainties satisfying specific constraints. Nevertheless, this method belongs to a point-estimation scheme and cannot provide interval bounds for estimation errors, and its assumptions on uncertainties are relatively restrictive. Reference [30] indicates that, for interval type-2 T-S fuzzy systems with disturbances and noises, traditional Luenberger observers inevitably suffer from large conservatism in set estimation, since disturbances and noises severely degrade the accuracy of set boundaries and further impair state and residual estimation. To address this issue, the unknown input observer is utilized for disturbance decoupling, while zonotopic analysis is adopted to tighten feasible sets and improve estimation performance. Reference [31] also develops a T-S fuzzy Luenberger observer modified from the conventional observer architecture. Weighted by T-S fuzzy membership functions for local linear sub-observers, it achieves joint estimation of system states and sensor attacks. Therefore, applying the set-membership method to T-S fuzzy models with uncertainties to obtain a feasible set of system states could be a meaningful alternative [32,33,34,35], with new applications for the solution of FDI problems already reported [36,37,38,39]. Reference [39] designs a fault-detection observer for time-delay T-S fuzzy singular systems based on finite-frequency $H_{-}$/$L_{\infty }$ indices. Zonotopic set-membership techniques are adopted to optimize thresholds and design conditions, which effectively reduces detection conservatism.

This paper discusses the optimization of a zonotopic set-membership construction method for uncertain T-S fuzzy system with actuator faults and sensor faults. Then, we attempt to solve FDI problems by constructing proper residuals based on the optimization of zonotopic set-membership construction. The main contributions of this paper are summarized as follows.

(1) This work develops a zonotopic set-membership estimation scheme for disturbed T-S fuzzy systems by intersecting state-predicted and measurement-updated zonotopes. In contrast with existing zonotope-based methods with single stability constraints, two quantitative indices are adopted to characterize zonotope conservatism. A parameterized correction matrix is further optimized via convex LMI constraints, which helps compress the enveloping zonotope volume and reduce estimation redundancy.
(2) Based on the optimized zonotopic estimation, the bounds of system states and outputs are explicitly derived. Instead of adopting fixed artificial thresholds in conventional FDI approaches, real-time dynamic residual thresholds are extracted from optimized output intervals. This strategy can mitigate false alarms caused by time-varying disturbances and improve fault-detection robustness.
(3) To isolate actuator and sensor faults under coupled fault conditions, two independent zonotopic estimation branches are constructed by treating different faults as equivalent unknown disturbances. In contrast to traditional single-residual detection frameworks, two sets of correction matrices and dedicated isolation residuals are established, which enables effective discrimination between actuator and sensor faults.

To more intuitively reveal the differences between the proposed method and several representative existing approaches, references [10,28,30] are selected for qualitative comparisons along four dimensions—interval estimation, fault detection, fault isolation and computational cost—as shown in Table 1. Overall, compared with the existing methods reported in Refs. [10,28,30], the proposed scheme achieves lower estimation conservatism, improved anti-interference fault detection, and effective coupled fault isolation, exhibiting better comprehensive applicability. However, the dual-branch optimization design inevitably increases computational burden, and the performance for strongly coupled fault scenarios remains to be further optimized in future work.

The structure of this paper is arranged as follows: The system model and foundational knowledge of zonotope theory, including core concepts and essential properties, are presented in Section 2. Section 3 discusses the issues of the optimization of zonotopic set-membership construction and fault detection. Section 4 completes the fault-isolation design for sensors and actuators. In Section 5, a simulation example is investigated to verify the feasibility and effectiveness of the proposed scheme. Finally, Section 6 concludes the research findings of this paper.

## 2. Preliminaries

The considered T-S fuzzy system can be presented by fuzzy IF–THEN rules as follows:

Plant Rule *i*: If $z_{1,k}$ is ${\tilde{F}}_{i1}$ and $z_{2,k}$ is ${\tilde{F}}_{i2}$ and … and $z_{\Lambda ,k}$ is ${\tilde{F}}_{i\Lambda }$, THEN

$$\left\{\begin{array}{c} x_{k+1}=A_{i}x_{k}+B_{i}\left(u_{k}+f_{a,k}\right)+D_{i}w_{k}\\ y_{k}=Cx_{k}+Fv_{k}+Hf_{s,k} \end{array}\right.\;i\in N=\left\{1,\ldots ,N\right\}$$

where $x_{k}\in R^{n},\;u_{k}\in R^{m},\;y_{k}\in R^{p},\;w_{k}\in R^{w},\;v_{k}\in R^{v},\;f_{a,k}\in R^{m},\;f_{s,k}\in R^{r}$ are the vector values at time *k* of the state, control input, output, external disturbance, measurement noise, actuator fault and sensor fault, respectively. $z_{j,k}$ (j=1,…,Λ) is the premise variable, ${\tilde{F}}_{ij}$ is a fuzzy set defined by the membership function, i.e., $\mu_{ij}\left(z_{k}\right)$ (i=1,…,N;j=1,…,Λ), where $z_{k}={\left[\begin{array}{ccc} z_{1,k}^{T} & \ldots & z_{\Lambda ,k}^{T} \end{array}\right]}^{T}$ represents the premise variable vector at time *k*. $A_{i}$, $B_{i}$, $D_{i}$ (i=1,…,N), *C*, *F* and *H* are constant matrices with compatible dimensions. Then, the T-S fuzzy model can be inferred by

(1) $$\left\{\begin{array}{c} x_{k+1}=A\left(h_{k}\right)x_{k}+B\left(h_{k}\right)\left(u_{k}+f_{a,k}\right)+D\left(h_{k}\right)w_{k}\\ y_{k}=Cx_{k}+Fv_{k}+Hf_{s,k} \end{array}\right.$$

where $A\left(h_{k}\right)=\sum_{i=0}^{N}h_{i}\left(z_{k}\right)A_{i},\;B\left(h_{k}\right)=\sum_{i=0}^{N}h_{i}\left(z_{k}\right)B_{i},\;D\left(h_{k}\right)=\sum_{i=0}^{N}h_{i}\left(z_{k}\right)D_{i},\;$ with $h_{i}\left(z_{k}\right)=\frac{\prod_{j=1}^{\Lambda }\mu_{ij}\left(z_{k}\right)}{\sum_{i=1}^{N}\prod_{j=1}^{\Lambda }\mu_{ij}\left(z_{k}\right)}.$ Obviously, we have $0\leq h_{i}\left(z_{k}\right)\leq 1\;\left(i=1,\ldots ,N\right)$ and $\sum_{i=1}^{N}h_{i}\left(z_{k}\right)=1$ because of $0\leq \mu_{ij}\left(z_{k}\right)\leq 1\;\left(i=1,\ldots ,N;j=1,\ldots ,\Lambda \right).$

**Assumption 1.** 
*The initial state ($x_{0}$), the external disturbance ($w_{k}$) and the measurement noise ($v_{k}$) are all unknown but bounded, satisfying ${\underline{x}}_{0}\leq x_{0}\leq {\bar{x}}_{0},\;\underline{w}\leq w_{k}\leq \bar{w},\;\mathrm{and}\;\underline{v}\leq v_{k}\leq \bar{v},$ where ${\underline{x}}_{0}$, ${\bar{x}}_{0}$, $\underline{w}$, $\bar{w}$, $\underline{v}$ and $\bar{v}$ are all known constant vectors. Moreover, when faults occur, they are bounded with ${\underline{f}}_{a}\leq f_{a,k}\leq {\bar{f}}_{a},\;\mathrm{and}\;{\underline{f}}_{s}\leq f_{s,k}\leq {\bar{f}}_{s},$ where ${\underline{f}}_{a}$, ${\bar{f}}_{a}$, ${\underline{f}}_{s}$ and ${\bar{f}}_{s}$ are known vectors.*

**Definition 1.** 
*An n-dimensional s-order zonotope (Z) is constructed via the affine transformation of the unit hypercube $B^{s}={\left[-1,1\right]}^{s}$ where s≥n: $Z=p⊕HB^{s}=\left\{p+Hz|z\in B^{s}\right\},$ in which the $p\in R^{n}$ vector refers to the central point of zonotope Z, while $H\in R^{n\times s}$ is the generator matrix corresponding to the zonotope. The operator (*⊕*) used in the formula denotes the Minkowski sum in geometric mathematics.*

To streamline the symbolic expression of zonotopes, we adopt the simplified form, i.e., Z=〈p,H〉, to characterize a standard zonotope. On the basis of the above definition, a set of fundamental properties of zonotopes can be concluded as follows.

**Property 1.** 
$$\langle p_{1},H_{1}\rangle ⊕\langle p_{2},H_{2}\rangle =\langle p_{1}+p_{2},\left[H_{1}\;H_{2}\right]\rangle$$

L⊙〈p,H〉⊕〈Lp,LH〉

*where $p,p_{1},p_{2}\in R^{n}$, $H_{1}\in R^{n\times s_{1}}$, $H_{2}\in R^{n\times s_{2}}$, $H\in R^{n\times s}$, and $L\in R^{l\times n}$. Here, *∘* stands for a linear map.*

**Property 2.** 
*Let Z be a zonotope such that $Z=p⊕HB^{s}\subset R^{n}$. We reorder all columns of matrix H by their Euclidean norms in descending sequence, and the reordered matrix is denoted by $\hat{H}$. For any integer (d) in the range of n≤d≤s, the zonotope (Z) is contained in $\langle p,R_{d}\left(H\right)\rangle$. The matrix ($R_{d}\left(H\right)=\left[H^{a}\;H^{b}\right]\in R^{n\times d}$) is composed of two parts. The first part ($H^{a}$) takes the first d−n columns from $\hat{H}$, and the second part ($H^{b}$) is a diagonal matrix. Each diagonal entry of $H^{b}$ is calculated as $H_{i,i}^{b}=\sum_{j=d-n+1}^{s}\left|{\hat{H}}_{ij}\right|\;\left(i=1,\ldots ,n\right)$ where ${\hat{H}}_{ij}$ is the element on the i-th row and j-th column of $\hat{H}$.*

**Property 3.** 
*Consider an n-dimensional zonotope of order s, expressed as $Z=p⊕HB^{s}\subset R^{n}$. Take any vector ($x={\left[\begin{array}{ccc} x_{1} & \cdots & x_{n} \end{array}\right]}^{T}$) inside Z. Each component of x satisfies the bound expressed as*

$$p_{i}-\sum_{j=1}^{s}|H_{ij}|\;\leq x_{i}\leq p_{i}+\sum_{j=1}^{s}\left|H_{ij}\right|\;\left(i=1,\ldots ,n\right).$$

*In this expression, $H_{ij}$ stands for the (i,j)-th element of matrix H. Meanwhile, the vector expressed as $p={\left[\begin{array}{ccc} p_{1} & \cdots & p_{n} \end{array}\right]}^{T}\in R^{n}$ is defined as the center of zonotope Z.*

**Property 4.** 
*For any $x\in R^{n}$: $\underline{x}\leq x\leq \bar{x},$ where $\underline{x},\bar{x}\in R^{n}$ and $x\in Z_{x}=p_{x}⊕H_{x}B^{s}=\langle p_{x},H_{x}\rangle ,$ where $p_{x}=\frac{1}{2}\left(\bar{x}+\underline{x}\right)\;\mathrm{and}\;H_{x}=\frac{1}{2}\;\mathrm{diag}\left(\bar{x}-\underline{x}\right).$*

**Lemma 1.** 
*Under Assumption 1, we have $x_{0}\in X_{x_{0}}=\langle p_{x_{0}},H_{x_{0}}\rangle ,\;w_{k}\in W=\langle p_{w},H_{w}\rangle ,\;v_{k}\in V=\langle p_{v},H_{v}\rangle ,\;u_{k}\in U=\langle p_{u_{k}},0\rangle ,$ where $p_{x_{0}}=\frac{1}{2}\left({\bar{x}}_{0}+x_{0}\right),\;H_{x_{0}}=\frac{1}{2}\mathrm{diag}\left({\bar{x}}_{0}-x_{0}\right),\;$$p_{w}=\frac{1}{2}\left(\bar{w}+w\right),\;H_{w}=\frac{1}{2}\mathrm{diag}\left(\bar{w}-w\right),\;p_{v}=\frac{1}{2}\left(\bar{v}+v\right),\;H_{v}=\frac{1}{2}\mathrm{diag}\left(\bar{v}-v\right),\;p_{u_{k}}=u_{k}.$*

**Proof.**  The proof can be finished directly by using Property 4.    □

**Definition 2.** 
*Since for any k≥0, $w_{k}\in W$ and $v_{k}\in V$, the worst-case $w_{k}$ and $v_{k}$ on the boundary of $W=\langle p_{w},H_{w}\rangle \;\mathrm{and}\;V=\langle p_{v},H_{v}\rangle$ are given by $\max_{b\in B^{n_{w}}}∥H_{w}b∥\;\mathrm{and}\;\max_{b\in B^{n_{v}}}∥H_{v}b∥.$ The $L_{\infty }$ norm of $w_{k}$ and $v_{k}$ are denoted by*

$$\begin{array}{cc} {∥w∥}_{\infty } & =\underset{k}{\sup}|w_{k}|=\max_{b\in B^{n_{w}}}∥H_{w}b∥\\ {∥v∥}_{\infty } & =\underset{k}{\sup}|v_{k}|=\max_{b\in B^{n_{v}}}∥H_{v}b∥. \end{array}$$

## 3. Zonotopic Set-Membership Construction and Fault Detection

Within this part of the work, we aim to put forward a construction strategy for zonotopic set-membership that can be utilized to acquire interval estimates of system states. Subsequently, relying on the acquired state-interval estimation results, a residual building algorithm is proposed for the purpose of fault detection.

### 3.1. Zonotopic Set-Membership Construction

Generally, the zonotopic set-membership construction contains three steps: prediction, measurement, and correction.

**Lemma 2.** 
*Assume the actuator fault $f_{a,k}$ and sensor fault $f_{s,k}$ are identically zero. Under the assumption of $f_{a,k}\equiv 0$ and $f_{s,k}\equiv 0$, we have $x_{k}\in X_{x_{k}}=\langle p_{x_{k}},H_{x_{k}}\rangle ,Cx_{k}\in X_{Cx_{k}}=\langle y_{k}-Fp_{v},-FH_{v}\rangle \;\;\left(k=0,1,2,\ldots \right),$ where*

(2) $$\begin{array}{cc} p_{x_{k}} & =A\left(h_{k-1}\right)p_{x_{k-1}}+B\left(h_{k-1}\right)u_{k-1}+D\left(h_{k-1}\right)p_{w}\\ H_{x_{k}} & =\left[A\left(h_{k-1}\right)H_{x_{k-1}}\;D\left(h_{k-1}\right)H_{w}\right] \end{array}\;\left(k=1,2,\ldots \right)$$

*where $p_{x_{0}}$, $H_{x_{0}}$, $p_{v}$, $H_{v}$, $p_{w}$ and $H_{w}$ are defined in Lemma 1.*

**Proof.**  Firstly, according to Lemma 1, $x_{0}\in X_{x_{0}}=\langle p_{x_{0}},H_{x_{0}}\rangle .$ Also notice that $Cx_{0}=y_{0}-Fv_{0}$; thus, $Cx_{0}\in \langle y_{0},0\rangle ⊕\left(-F\right)⊙\langle p_{v},H_{v}\rangle =\langle y_{0}-Fp_{v},-FH_{v}\rangle .$ Therefore, when k=0, the result of Lemma 2 is true. Secondly, suppose that for *k*, the conclusion of Lemma 2 is true. Then, for the case of k+1, applying Property 1 on (1) gives

$$\begin{array}{cc} x_{k+1} & =A\left(h_{k}\right)x_{k}+B\left(h_{k}\right)u_{k}+D\left(h_{k}\right)w_{k}\\ \; & \in A\left(h_{k}\right)\langle p_{x_{k}},H_{x_{k}}\rangle ⊕B\left(h_{k}\right)\langle p_{u_{k}},0\rangle ⊕D\left(h_{k}\right)\langle p_{w},H_{w}\rangle \\ \; & =\langle A\left(h_{k}\right)p_{x_{k}},A\left(h_{k}\right)H_{x_{k}}\rangle ⊕\langle B\left(h_{k}\right)p_{u_{k}},0\rangle ⊕\langle D\left(h_{k}\right)p_{w},D\left(h_{k}\right)H_{w}\rangle \\ \; & =\langle A\left(h_{k}\right)p_{x_{k}}+B\left(h_{k}\right)p_{u_{k}}+D\left(h_{k}\right)p_{w},\;\left[A\left(h_{k}\right)H_{x_{k}}\;D\left(h_{k}\right)H_{w}\right]\rangle \end{array}$$

Based on definition (2), we know that $x_{k+1}\in X_{x_{k+1}}=\langle p_{x_{k+1}},H_{x_{k+1}}\rangle .$

Similarly, from the second equation, we have $Cx_{k+1}=y_{k+1}-Fv_{k+1}\in \langle y_{k+1},0\rangle ⊕\left[-F\langle p_{v},H_{v}\rangle \right]=\langle y_{k+1}-Fp_{v},-FH_{v}\rangle ,$ which implies that $Cx_{k+1}\in X_{Cx_{k+1}}$. As a result, Lemma 2 is proven by the principle of mathematical induction.    □

The formulation ($H_{x_{k}}$) given in (2) indicates that the dimension of $H_{x_{k}}$ will increase as the time (*k*) increases, which leads to the increasing of the order of $x_{k}\in X_{k}=\langle p_{x_{k}},H_{x_{k}}\rangle$. Consequently, as the time (*k*) increases, the order of $X_{{\hat{x}}_{k}}=X_{x_{k}}\cap X_{Cx_{k}}$ increases. To avoid the dimension explosion problem, we perform a model order-reduction operation based on Property 2.

$H_{{\hat{x}}_{k}}$ denotes $R_{d}\left(H_{x_{k}}\right)$. Now, because $x_{k}\in X_{k}=\langle p_{x_{k}},H_{x_{k}}\rangle \subseteq \langle p_{x_{k}},R_{d}\left(H_{x_{k}}\right)\rangle$ and $Cx_{k}\in X_{Cx_{k}}=\langle y_{k}-Fp_{v},-FH_{v}\rangle$, there exist two unitary vectors ($z_{1}\in R^{d}$ and $z_{2}\in R^{v}$, with *d* being the number of columns of the reduced-order matrix ($R_{d}\left(H_{{\hat{x}}_{k}}\right)$) such that

(3) $$\begin{array}{cc} x_{k} & =A\left(h_{k-1}\right)p_{x_{k-1}}+B\left(h_{k-1}\right)u_{k-1}+D\left(h_{k-1}\right)p_{w}+H_{{\hat{x}}_{k}}z_{1}\\ \; & =A\left(h_{k-1}\right)p_{x_{k-1}}+B\left(h_{k-1}\right)u_{k-1}+D\left(h_{k-1}\right)p_{w}\\ \; & \;+\left[A\left(h_{k-1}\right)H_{x_{k-1}}\;D\left(h_{k-1}\right)H_{w}\right]z_{1} \end{array}$$

and $Cx_{k}=y_{k}-Fp_{v}-FH_{v}z_{2},$ which is just

(4) $$y_{k}=Cx_{k}+Fp_{v}+FH_{v}z_{2}.$$

On one hand, for any matrix ($\Lambda \left(h_{k-1}\right)\in R^{n\times p}$), (3) can be rewritten as

(5) $$\begin{array}{cc} x_{k} & =A\left(h_{k-1}\right)p_{x_{k-1}}+B\left(h_{k-1}\right)u_{k-1}+D\left(h_{k-1}\right)p_{w}\\ \; & \;+\Lambda \left(h_{k-1}\right)CH_{{\hat{x}}_{k}}z_{1}+\left(I_{n}-\Lambda \left(h_{k-1}\right)C\right)H_{{\hat{x}}_{k}}z_{1} \end{array}$$

On the other hand, substituting (3) into (4) gives

(6) $$y_{k}=CA\left(h_{k-1}\right)p_{x_{k-1}}+CB\left(h_{k-1}\right)u_{k-1}+CD\left(h_{k-1}\right)p_{w}+Fp_{v}+CH_{{\hat{x}}_{k}}z_{1}+FH_{v}z_{2}$$

Equation (6) gives

(7) $$CH_{{\hat{x}}_{k}}z_{1}=y_{k}-CA\left(h_{k-1}\right)p_{x_{k-1}}-CB\left(h_{k-1}\right)u_{k-1}-CD\left(h_{k-1}\right)p_{w}-Fp_{v}-FH_{v}z_{2}$$

By substituting (7) into (5) and using the second equation of (2), we can obtain

(8) $$\begin{array}{cc} x_{k} & =\left(I_{n}-\Lambda \left(h_{k-1}\right)C\right)A\left(h_{k-1}\right)\left(p_{x_{k-1}}+B\left(h_{k-1}\right)u_{k-1}+D\left(h_{k-1}\right)p_{w}\right)\\ \; & \;+\;\Lambda \left(h_{k}\right)y_{k}-\Lambda \left(h_{k}\right)Fp_{v}\\ \; & \;+\left[\left(I_{n}-\Lambda \left(h_{k-1}\right)C\right)A\left(h_{k-1}\right)H_{x_{k-1}}\;\left(I_{n}-\Lambda \left(h_{k-1}\right)C\right)D\left(h_{k-1}\right)H_{w}\;-\Lambda \left(h_{k-1}\right)FH_{v}\right]z \end{array}$$

where $z={\left[\begin{array}{cc} z_{1}^{T} & z_{2}^{T} \end{array}\right]}^{T}.$

$X_{{\hat{x}}_{k}}=X_{x_{k}}\cap X_{Cx_{k}}$ denotes a zonotope of the estimation of state $x_{k}$, that is, any ${\hat{x}}_{k}\in X_{{\hat{x}}_{k}}$, ${\hat{x}}_{k}$ can act as an estimation of $x_{k}$. Therefore, by referring to (8), we have

(9) $$X_{{\hat{x}}_{k}}=\langle p_{{\hat{x}}_{k}},H_{{\hat{x}}_{k}}\rangle$$

where

(10) $$\begin{array}{cc} p_{{\hat{x}}_{k}} & =\left(I_{n}-\Lambda \left(h_{k-1}\right)C\right)A\left(h_{k-1}\right)\left(p_{{\hat{x}}_{k-1}}+B\left(h_{k-1}\right)u_{k-1}+D\left(h_{k-1}\right)p_{w}\right)\\ \; & \;+\;\Lambda \left(h_{k-1}\right)y_{k}-\Lambda \left(h_{k-1}\right)Fp_{v}\\ H_{{\hat{x}}_{k}} & =\left[\left(I_{n}-\Lambda \left(h_{k-1}\right)C\right)A\left(h_{k-1}\right)H_{{\hat{x}}_{k-1}}\;\left(I_{n}-\Lambda \left(h_{k-1}\right)C\right)D\left(h_{k-1}\right)H_{w}\right.\\ \; & \;\left.\;-\Lambda \left(h_{k-1}\right)FH_{v}\right] \end{array}$$

From the foregoing analysis, we derive the core theoretical conclusion presented below.

**Theorem 1.** 
*For the T-S fuzzy model (1), when $f_{a,k}\equiv 0$ and $f_{s,k}\equiv 0$, for some given correction matrix ($\Lambda \left(h_{k-1}\right)\in R^{n\times p}$), for any ${\hat{x}}_{k}\in X_{{\hat{x}}_{k}}=\langle p_{{\hat{x}}_{k}},H_{{\hat{x}}_{k}}\rangle$, ${\hat{x}}_{k}$ can act as an estimation of $x_{k}$, where $p_{{\hat{x}}_{k}}$ and $H_{{\hat{x}}_{k}}$ are defined by (10).*

Since the intersection zonotope ($X_{{\hat{x}}_{k}}=\langle p_{{\hat{x}}_{k}},H_{{\hat{x}}_{k}}\rangle$) provides a bounded domain for all uncertain system states, a proper correction matrix ($\Lambda (h_{k-1})$) is formulated to restrict the expansion of this zonotope set. To evaluate and limit its geometric magnitude, two radius metrics are introduced to quantify the size of $X_{{\hat{x}}_{k}}$.

**Definition 3.** 
*The radius of the zonotope ($X_{{\hat{x}}_{k}}=\langle p_{{\hat{x}}_{k}},H_{{\hat{x}}_{k}}\rangle$) is defined as*

(11) $$\ell_{k}=\max_{{\hat{x}}_{k}\in X_{{\hat{x}}_{k}}}{∥{\hat{x}}_{k}-p_{{\hat{x}}_{k}}∥}^{2}=\max_{b\in B}{∥H_{{\hat{x}}_{k}}b∥}^{2}$$

*and the P-radius is defined as*

(12) $$\rho_{k}=\max_{{\hat{x}}_{k}\in X_{{\hat{x}}_{k}}}{∥{\hat{x}}_{k}-p_{{\hat{x}}_{k}}∥}_{P}^{2}=\max_{b\in B}{∥H_{{\hat{x}}_{k}}b∥}_{P}^{2}$$

Based on the above definitions, the condition used to constrain $X_{{\hat{x}}_{k}}$ is given in the subsequent theorem.

**Theorem 2.** 
*Consider the T-S fuzzy descriptor model (1) and $X_{{\hat{x}}_{k}}$ in (9). If there exist a symmetric positive matrix ($P\in R^{n\times n}$), a parameter correction matrix ($\Lambda_{i}\in R^{n\times p}$) and a positive scalar (γ>0) that satisfy*

(13) $$\left[\begin{array}{cccc} -\alpha P & * & * & *\\ 0 & -(1-\alpha )\beta I & * & *\\ 0 & 0 & -(1-\alpha )(1-\beta )I & *\\ PA_{i}-P\Lambda_{i}CA_{j} & PD_{i}-P\Lambda_{i}CD_{j} & -P\Lambda_{i}F & -P \end{array}\right]<0,\;\left(i,j=1,\ldots ,N\right)$$

*and*

(14) $$\left[\begin{array}{cc} I_{n} & *\\ \gamma P & P \end{array}\right]<0$$

*consequently, zonotope $X_{{\hat{x}}_{k}}$ satisfies*

(15a) $$\rho_{k}\leq \alpha \rho_{k-1}+\left(1-\alpha \right)\vartheta_{w}$$

(15b) $$\ell_{k}\leq \gamma^{2}\rho_{k}$$

*where α,β∈(0,1) and*

(16) $$\vartheta_{w}=\max_{b\in B}\beta ∥H_{w}b_{1}{∥}^{2}+\max_{b\in B}\left(1-\beta \right){∥H_{v}b_{2}∥}^{2}$$

**Proof.**  According to (18) in Definition 2, (15a), (16) can become

(17) $$\begin{array}{cc} \max_{b\in X_{{\hat{x}}_{k}}}{∥H_{{\hat{x}}_{k}}b∥}_{P}^{2}\leq & \alpha \max_{\bar{b}\in X_{{\hat{x}}_{k-1}}}∥H_{{\hat{x}}_{k-1}}\bar{b}{∥}_{P}^{2}+\left(1-\alpha \right)\beta \max_{b_{1}\in B}{∥H_{w}b_{1}∥}^{2}\\ \; & +\left(1-\alpha \right)\left(1-\beta \right)\max_{b_{2}\in B}{∥H_{v}b_{2}∥}^{2} \end{array}$$

where $b={\left[\begin{array}{ccc} {\bar{b}}^{T} & b_{1}^{T} & b_{2}^{T} \end{array}\right]}^{T}.$ Therefore, a sufficient condition for (17) is

(18) $$\begin{array}{cc} b^{T}H_{{\hat{x}}_{k}}^{T}PH_{{\hat{x}}_{k}}b-\alpha {\bar{b}}^{T}H_{{\hat{x}}_{k-1}}^{T}PH_{{\hat{x}}_{k-1}}\bar{b} & -\left(1-\alpha \right)\beta b_{1}^{T}H_{w}^{T}H_{w}b_{1}\\ \; & \;-\left(1-\alpha \right)\left(1-\beta \right)b_{2}^{T}H_{v}^{T}H_{v}b_{2}<0 \end{array}$$

Substituting the $H_{{\hat{x}}_{k}}$ in (10) into (18) gives

$${\left[\begin{array}{c} \phi \\ \varphi \\ \psi \end{array}\right]}^{T}\left[\begin{array}{ccc} \Omega_{11} & \Omega_{12} & \Omega_{13}\\ \Omega_{21} & \Omega_{22} & \Omega_{23}\\ \Omega_{31} & \Omega_{32} & \Omega_{33} \end{array}\right]\left[\begin{array}{c} \phi \\ \varphi \\ \psi \end{array}\right]<0$$

where $\phi =H_{{\hat{x}}_{k-1}}\bar{b}$, $\varphi =H_{w}b_{1}$, $\psi =H_{v}b_{2}$, and

$$\begin{array}{cccc} & \Omega_{11}=A{\left(h_{k-1}\right)}^{T}{\left(I-\Lambda \left(h_{k-1}\right)C\right)}^{T}P\left(I-\Lambda \left(h_{k-1}\right)C\right)A\left(h_{k-1}\right)-\alpha P & & \\ & \Omega_{12}=A{\left(h_{k-1}\right)}^{T}{\left(I-\Lambda \left(h_{k-1}\right)C\right)}^{T}P\left(I-\Lambda \left(h_{k-1}\right)C\right)D\left(h_{k-1}\right) & & \\ & \Omega_{13}=-A{\left(h_{k-1}\right)}^{T}{\left(I-\Lambda \left(h_{k-1}\right)C\right)}^{T}P\Lambda \left(h_{k-1}\right)F & & \\ & \Omega_{21}=D{\left(h_{k-1}\right)}^{T}{\left(I-\Lambda \left(h_{k-1}\right)C\right)}^{T}P\left(I-\Lambda \left(h_{k-1}\right)C\right)A\left(h_{k-1}\right) & & \\ & \Omega_{22}=D{\left(h_{k-1}\right)}^{T}{\left(I-\Lambda \left(h_{k-1}\right)C\right)}^{T}P\left(I-\Lambda \left(h_{k-1}\right)C\right)D\left(h_{k-1}\right)-\left(1-\alpha \right)\beta I & & \\ & \Omega_{23}=-D{\left(h_{k-1}\right)}^{T}{\left(I-\Lambda \left(h_{k-1}\right)C\right)}^{T}P\Lambda \left(h_{k-1}\right)F & & \\ & \Omega_{31}=-F^{T}\Lambda {\left(h_{k-1}\right)}^{T}P\left(I-\Lambda \left(h_{k-1}\right)C\right)A\left(h_{k-1}\right) & & \\ & \Omega_{32}=-F^{T}\Lambda {\left(h_{k-1}\right)}^{T}P\left(I-\Lambda \left(h_{k-1}\right)C\right)D\left(h_{k-1}\right) & & \\ & \Omega_{33}=F^{T}\Lambda {\left(h_{k-1}\right)}^{T}P\Lambda \left(h_{k-1}\right)F-\left(1-\alpha \right)\left(1-\beta \right)I, & & \end{array}$$

yhat is, (21a) holds if

(19) $$\left[\begin{array}{ccc} \Omega_{11} & \Omega_{12} & \Omega_{13}\\ \Omega_{21} & \Omega_{22} & \Omega_{23}\\ \Omega_{31} & \Omega_{32} & \Omega_{33} \end{array}\right]<0$$

According to the Schur complement lemma, (19) is equivalent to

$$\left[\begin{array}{cccc} -\alpha P & * & * & *\\ 0 & -(1-\alpha )\beta I & * & *\\ 0 & 0 & -(1-\alpha )(1-\beta )I & *\\ P\left(I-\Lambda (h_{k-1})C\right)A\left(h_{k-1}\right)\; & P\left(I-\Lambda (h_{k-1})C\right)D\left(h_{k-1}\right)\; & -P\Lambda (h_{k-1})F\; & -P \end{array}\right]<0$$

which can be rewritten as

(20) $$\sum_{i=1}^{N}\sum_{j=1}^{N}h_{i}\left(z_{k-1}\right)h_{j}\left(z_{k-1}\right)\left[\begin{array}{cccc} -\alpha P & * & * & *\\ 0 & -(1-\alpha )\beta I & * & *\\ 0 & 0 & -(1-\alpha )(1-\beta )I & *\\ PA_{i}-P\Lambda_{i}CA_{j}\; & PD_{i}-P\Lambda_{i}CD_{j}\; & -P\Lambda_{i}F\; & -P \end{array}\right]<0.$$

(20) means (13). Furthermore, according to (17) in Definition 3, the radius parameter of the intersection zonotope ($X_{{\hat{x}}_{k}}$) at time *k* takes the form of $\ell_{k}=\max_{\hat{z}\in Z}{∥H_{{\hat{x}}_{k}}b∥}^{2}.$ Thus, from (15b), we derive $I-\gamma^{2}P\leq 0$. By virtue of the Schur complement lemma, we obtain (14). For the polytopic representation of the state matrix ($A(h_{k-1})$, $B(h_{k-1})$, $D(h_{k-1})$) and correction matrix ($\Lambda (h_{k-1})$), (20) may encounter the problem of double-sum computation. To restructure the double-sum problem, we utilize the result presented below.    □

**Lemma 3** 
([40])**.** *This work defines the double-sum condition as follows:*

(21) $$\Gamma \left(v_{k},v_{k}\right)=\sum_{i=1}^{r}\sum_{j=1}^{y}\mu_{i}\left(v_{k}\right)\mu_{j}\left(v_{k}\right)\Gamma_{i,j}>0$$

*Then, (21) holds true if the subsequent requirements are satisfied (∀i,j=1,…,r and i≠j):*

(22) $$\begin{array}{cc} \Gamma_{i,i} & >0\\ \frac{2}{r-1}\Gamma_{i,i}+\Gamma_{i,j}+\Gamma_{j,i} & >0 \end{array}$$

Applying Lemma 3, we convert (20) (with multiple vertices) into the forms of (21) and (22), as shown in the corollary below.

**Corollary 1.** 
*Consider the T-S fuzzy model (1) if there exists a symmetric positive matrix ($P\in R^{n\times n}$) and matrix ($Y_{i}\in R^{n\times p}$) such that*

(23) $$\begin{array}{cc} \Phi_{i,i} & >0,\;i=1,2,\ldots ,\lambda \\ \frac{2}{\lambda -1}\Phi_{i,i}+\Phi_{i,j}+\Phi_{j,i} & \geq 0,\;1\leq i<j\leq \lambda \end{array}$$

*where*

$$\Phi_{i,j}=\left[\begin{array}{cccc} \alpha P & * & * & *\\ 0 & (1-\alpha )\beta & * & *\\ 0 & 0 & \left(1-\alpha )(1-\beta \right) & *\\ -PA_{i}+Y_{j}CA_{i} & -PD_{i}+Y_{j}CD_{i} & Y_{j}F & P \end{array}\right]$$

*Then, (20) is satisfied.*

From Theorem 2, we can derive an adaptive upper bound for the radius of the intersection zonotope ($X_{{\hat{x}}_{k}}$) in the theorem below. $X_{{\hat{x}}_{k}}$ can be obtained in the following theorem.

**Theorem 3.** 
*The radius ($L_{\infty }$) performance of the intersecting zonotope ($X_{{\hat{x}}_{k}}$) in (3) at time k is captured by*

$$\ell_{k}\leq \gamma^{2}\alpha^{k}\rho_{0}+\gamma^{2}\left({\beta ∥w∥}_{\infty }^{2}+\left(1-\beta \right){∥v∥}_{\infty }^{2}\right)$$

*with $\rho_{0}=\max_{b_{0}\in B^{n_{0}}}{∥H_{0}b_{0}∥}_{P}^{2}.$*

**Proof.**  From Definition 1 and Theorem 2, we have

$$\ell_{w}=\max_{b\in B}\beta {∥H_{w}b∥}^{2}+\max_{b\in B}\left(1-\beta \right){∥H_{v}b∥}^{2}={\beta ∥w∥}_{\infty }^{2}+\left(1-\beta \right){∥v∥}_{\infty }^{2}.$$

From Theorem 2 $\left(\rho_{k}\leq \alpha \rho_{k-1}+\left(1-\alpha \right)\ell_{w}\right)$, for 0<α,β<1, through iterative operations, it can be obtained that

$$\begin{array}{cc} \rho_{k} & \leq \alpha \rho_{k-1}+\left(1-\alpha \right)\ell_{w}\\ & \leq \alpha \rho_{k-1}+\left(1-\alpha \right)\left({\beta ∥w∥}_{\infty }^{2}+\left(1-\beta \right){∥v∥}_{\infty }^{2}\right)\\ & \leq \alpha \left[\alpha \rho_{k-1}+\left(1-\alpha \right)\left({\beta ∥w∥}_{\infty }^{2}+\left(1-\beta \right){∥v∥}_{\infty }^{2}\right)\right]\\ & \;+\left(1-\alpha \right)\left({\beta ∥w∥}_{\infty }^{2}+\left(1-\beta \right){∥v∥}_{\infty }^{2}\right)\\ & \leq \cdots \\ & \leq \alpha^{k}\rho_{0}+\sum_{i=0}^{k-1}\alpha^{i}{\left(1-\alpha \right)\beta ∥w∥}_{\infty }^{2}+\sum_{i=0}^{k-1}\alpha^{i}\left(1-\alpha \right)\left(1-\beta \right){∥v∥}_{\infty }^{2}\\ & \leq \alpha^{k}\rho_{0}+\left(1-\alpha^{k}\right){\beta ∥w∥}_{\infty }^{2}+\left(1-\alpha^{k}\right)\left(1-\beta \right){∥v∥}_{\infty }^{2}\\ & \leq \alpha^{k}\rho_{0}+{\beta ∥w∥}_{\infty }^{2}+\left(1-\beta \right){∥v∥}_{\infty }^{2}. \end{array}$$

Therefore, from Theorem 2 $\left(\ell_{k}\leq \gamma^{2}\rho_{k}\right)$, we have

(24) $$\ell_{k}\leq \gamma^{2}\rho_{k}\leq \gamma^{2}\left(\alpha^{k}\rho_{0}+{\beta ∥w∥}_{\infty }^{2}+\left(1-\beta \right){∥v∥}_{\infty }^{2}\right)$$

   □

**Remark 1.** 
*It is worth mentioning that Theorem 2 offers a modified approach to acquire the zonotope formed by the intersection between the state set ($X_{k}$) and set Cx—namely, $X_{cx}$. The $\ell_{k}$ parameter is defined to quantify the scale of the generated zonotope, and its value complies with the constraint (15b) derived from Theorem 2. In addition, Theorem 3 establishes a time-varying upper bound for the aforementioned radius under the worst-case disturbance scenarios. As demonstrated in the proof process of Theorem 3, Formula (24) characterizes the correlation between this worst-case bound and the boundary value calculated by Theorem 2. This inequality indicates that the time-varying radius ($\ell_{k}$) is constrained by multiple factors, including the initial condition parameter ($\rho_{0}$), worst-case disturbances, the preset scalar (α∈(0,1)), and the positive scalar (γ>0).*

As the time step (*k*) accumulates, the value of the exponential term ($\alpha^{k}$) asymptotically approaches zero. For any sufficiently large integer ($k_{M}$), the worst-case boundary of $\ell_{k}$ can be deduced under extreme disturbance conditions for all time steps satisfying $k\geq k_{M}$, which is expressed as $\ell_{k}\leq \gamma^{2}\left({\beta ∥w∥}_{\infty }^{2}+\left(1-\beta \right){∥v∥}_{\infty }^{2}\right)\;\mathrm{for}\;\gamma >0.$

According to the theoretical deductions of Remark 1, we can calculate the optimal polytopic correction matrices ($\Lambda_{i}$) (i=1,…,h) through the solution to the subsequent optimization formulation:

(25) $$\min_{P,Y_{i}}\gamma$$

With constraints (13), (14), (15) and (23) imposed, the solution yields the tightest attainable worst-case bound for $\ell_{k}$ with minimal conservatism.

When α,β∈(0,1) are given, the constraints in (24) are linear and convex. γP term in (14) can be managed by solving the optimization problem (25) using a linear programming solver alongside line search for the minimum γ. Therefore, the optimal solutions of the optimization problem (25) are given as $\Lambda_{i}^{*}=P^{*-1}Y_{i}^{*}$ for i=1,…,N.

It should be noted that the optimization weights (α and β) must satisfy the convex feasible constraints of linear matrix inequalities to guarantee the stability of the observer. These weights can be determined empirically according to the noise and disturbance characteristics of practical systems, and parameter traversal simulations are adopted to verify the rationality of the empirical values.

In addition, although the proposed method involves LMI solutions and recursive zonotopic calculations, these two computations are decoupled. The LMI-based convex optimization is solved only once offline and consumes no online resources. The online part only implements lightweight zonotopic matrix iterations. $n_{x}$, $n_{y}$ and *N* denote the state dimension, output dimension and number of T-S fuzzy rules. The asymptotic online complexity is $O(Nn_{x}^{2})$, which theoretically reveals that the online burden grows linearly with the fuzzy-rule number (*N*). This work focuses on numerical simulation studies, and experimental measurement of actual running time is not performed. Nevertheless, the computational overhead rises with the state dimension ($n_{x}$), which is a common limitation of zonotope-based set-membership estimation.

### 3.2. Fault Detection Based on Zonotopic Set-Membership

We first define ${\hat{x}}_{k}={\left[\begin{array}{ccc} {\hat{x}}_{1,k} & \cdots & {\hat{x}}_{n,k} \end{array}\right]}^{T},\;{\hat{p}}_{{\hat{x}}_{k}}={\left[\begin{array}{ccc} {\hat{p}}_{1,{\hat{x}}_{k}} & \cdots & {\hat{p}}_{n,{\hat{x}}_{k}} \end{array}\right]}^{T},\;{\hat{H}}_{{\hat{x}}_{k}}={\left[\begin{array}{ccc} {\hat{H}}_{1,{\hat{x}}_{k}}^{T} & \cdots & {\hat{H}}_{n,{\hat{x}}_{k}}^{T} \end{array}\right]}^{T}.$ Then, according to Property 3, we have ${\underline{x}}_{k}\leq x_{k}\leq {\bar{x}}_{k},$ where ${\bar{x}}_{k}={\left[\begin{array}{ccc} {\bar{x}}_{1,k} & \cdots & {\bar{x}}_{n,k} \end{array}\right]}^{T}\;\mathrm{and}\;{\underline{x}}_{k}={\left[\begin{array}{ccc} {\underline{x}}_{1,k} & \cdots & {\underline{x}}_{n,k} \end{array}\right]}^{T}$ with ${\underline{x}}_{j,k}={\hat{p}}_{j,{\hat{x}}_{k}}-R_{d}\left({\hat{H}}_{j,{\hat{x}}_{k}}\right),\;{\bar{x}}_{j,k}={\hat{p}}_{j,{\hat{x}}_{k}}+R_{d}\left({\hat{H}}_{j,{\hat{x}}_{k}}\right).$ Here, $R_{d}\left({\hat{H}}_{j,{\hat{x}}_{k}}\right)$ returns the *j*th diagonal element of $R_{d}\left({\hat{H}}_{{\hat{x}}_{k}}\right)$. Now, we define

(26) $$\left\{\begin{array}{c} {\bar{y}}_{k}=C^{+}{\bar{x}}_{k}-C^{-}{\underline{x}}_{k}+F^{+}\bar{v}-F^{-}\underline{v}\\ {\underline{y}}_{k}=C^{+}{\underline{x}}_{k}-C^{-}{\bar{x}}_{k}+F^{+}\underline{v}-F^{-}\bar{v}, \end{array}\right.$$

and we have ${\underline{y}}_{k}\leq y_{k}\leq {\bar{y}}_{k}\;\left(k\geq 0\right)$ when there are no faults in the system. Based on the output interval estimation results in (26), a new residual design scheme is proposed to detect faults occurring in sensors and actuators. To begin with, we define two error terms: ${\bar{e}}_{y}=y-\bar{y}\;\mathrm{and}\;{\underline{e}}_{y}=\underline{y}-y.$ We then calculate two maximum values as ${\bar{e}}_{y,\max}=\max_{l\in \{1,\ldots ,p\}}\left({\bar{e}}_{y,l}\right)\;\mathrm{and}\;{\underline{e}}_{y,\max}=\max_{l\in \{1,\ldots ,p\}}\left({\underline{e}}_{y,l}\right),$ where the ${\bar{e}}_{y,l}$ and ${\underline{e}}_{y,l}$ symbols refer to the *l*-th element of vectors ${\bar{e}}_{y}$ and ${\underline{e}}_{y}$. The residual used for fault detection is constructed as $r_{d,k}=\max\left({\bar{e}}_{y,\max},{\underline{e}}_{y,\max}\right)$. Finally, the decision criteria for fault detection are presented below.

(27) $$r_{d,k}=\left\{\begin{array}{cc} >r_{0}, & \mathrm{fault}\;\mathrm{occurrence}\\ <r_{0}, & \mathrm{no}\;\mathrm{fault}\;\mathrm{occurrence} \end{array}\right.$$

where $r_{0}>0$ is a scalar called the fault detection threshold, which should be reasonably chosen, preliminarily set from engineering experience of system noise and calibrated by the maximum fault-free residual to avoid false alarms.

**Remark 2.** 
*It is necessary to distinguish two threshold-related concepts to avoid confusion. On one hand, the positive scalar ($r_{0}$) in Equation (27) is a fixed residual amplitude margin for binary fault judgment: if the constructed residual ($r_{d,k}$) exceeds $r_{0}$, a fault is declared; otherwise, the system operates under fault-free conditions. On the other hand, the so-called adaptive dynamic threshold proposed in this work does not refer to the constant ($r_{0}$) but the time-varying fault-free output envelope ($\left[{\underline{y}}_{k},{\bar{y}}_{k}\right]$) obtained by recursive zonotopic set-membership estimation. This output interval updates adaptively at each sampling instant with system states, measurement noise and external disturbances, and the residual signal ($r_{d,k}$) is generated based on this real-time interval. By optimizing the correction matrix via LMIs to reduce the conservatism of the zonotope envelope, the constructed residual can naturally adapt to time-varying uncertainties, which brings stronger robustness against noise while maintaining favorable fault-detection sensitivity. The fixed threshold ($r_{0}$) is empirically tuned slightly higher than the maximum residual amplitude under fault-free conditions with a proper safety margin. The detection performance is sensitive to the threshold value: a smaller $r_{0}$ tends to trigger false alarms, while a larger $r_{0}$ may reduce fault-detection sensitivity. For higher noise levels, a larger threshold is required to guarantee detection robustness.*

## 4. Fault Isolation

In this section, we plan to develop schemes of fault isolation between actuator and sensor faults.

$$\left\{\begin{array}{c} x_{k+1}=A_{i}x_{k}+B_{i}\left(u_{k}+f_{a,k}\right)+D_{i}w_{k}\\ y_{k}=Cx_{k}+Fv_{k}+Hf_{s,k} \end{array}\right.\;i\in N=\left\{1,\ldots ,N\right\}$$

### 4.1. Actuator Fault Isolation Strategy

The method of actuator fault isolation can be basically proposed in a way similar to that for fault detection.

When $f_{a,k}=0$ and $f_{s,k}\neq 0$, we have ${\underline{y}}_{a,k}\leq y_{k}\leq {\bar{y}}_{a,k}\;\left(k\geq 0\right),$ where

$$\left\{\begin{array}{c} {\bar{y}}_{a,k}=C^{+}{\bar{x}}_{k}-C^{-}{\underline{x}}_{k}+F^{+}\bar{v}-F^{-}\underline{v}+H^{+}{\bar{f}}_{sk}-H^{-}{\underline{f}}_{sk}\\ {\underline{y}}_{a,k}=C^{+}{\underline{x}}_{k}-C^{-}{\bar{x}}_{k}+F^{+}\underline{v}-F^{-}\bar{v}+H^{+}{\underline{f}}_{sk}-H^{-}{\bar{f}}_{sk} \end{array}\right.$$

Similar to the process obtained from (27), we construct a residual ($r_{a,k}$) as follows:

(28) $$r_{a,k}=\left\{\begin{array}{cc} >r_{a,0}, & \mathrm{actuator}\;\mathrm{fault}\;\mathrm{occurrence}\\ <r_{a,0}, & \mathrm{no}\;\mathrm{actuator}\;\mathrm{fault}\;\mathrm{occurrence} \end{array}\right.$$

where the positive scalar ($r_{a,0}$) is introduced as the isolation threshold, which requires a reasonable setting.

### 4.2. Sensor Fault Isolation Strategy

Firstly, under Assumption 1 and by using Property 4, we have $f_{a,k}\in F_{a}=\langle p_{f_{a,k}},H_{f_{a,k}}\rangle$, where $p_{a,k}=\frac{1}{2}\left({\underline{f}}_{a}+{\bar{f}}_{a}\right),\;H_{f_{a,k}}=\frac{1}{2}\mathrm{diag}\left({\bar{f}}_{a}-{\underline{f}}_{a}\right).$ Secondly, we set $\varphi_{k}={\left[\begin{array}{cc} f_{a,k}^{T} & w_{k}^{T} \end{array}\right]}^{T}$ and correspondingly denote $T\left(h_{k}\right)=\sum_{i=0}^{N}h_{i}\left(z_{k}\right)\left[\begin{array}{cc} B_{i} & D_{i} \end{array}\right],$ so the system (1) can be rewritten as

(29) $$\left\{\begin{array}{c} x_{s,k+1}=A\left(h_{k}\right)x_{s,k}+B\left(h_{k}\right)u_{k}+T\left(h_{k}\right)\varphi_{k}\\ y_{s,k}=Cx_{s,k}+Fv_{k}+Hf_{s,k}. \end{array}\right.$$

Obviously, $\underline{\varphi }\leq \varphi_{k}\leq \bar{\varphi },$ where $\bar{\varphi }={\left[\begin{array}{cc} {\bar{f}}_{a}^{T} & {\bar{w}}^{T} \end{array}\right]}^{T}\;\mathrm{and}\;\underline{\varphi }={\left[\begin{array}{cc} {\underline{f}}_{a}^{T} & {\underline{w}}^{T} \end{array}\right]}^{T}.$ Moreover, we have $\varphi_{k}\in \langle p_{\varphi_{k}},H_{\varphi_{k}}\rangle ,$ where $p_{\varphi_{k}}=\frac{1}{2}\left(\bar{\varphi }+\underline{\varphi }\right),\;H_{\varphi_{k}}=\frac{1}{2}\mathrm{diag}\left(\bar{\varphi }-\underline{\varphi }\right).$ Similarly, we have $x_{s,k}\in X_{x_{s,k}}=\langle p_{x_{s,k}},H_{x_{s,k}}\rangle$ and $Cx_{s,k}\in X_{Cx_{s,k}}=\langle y_{s,k}-Fp_{v},-FH_{v}\rangle$ (k=0,1,2,…), where $p_{x_{s,k}}=A\left(h_{k-1}\right)p_{x_{s,k-1}}+B\left(h_{k-1}\right)u_{k-1}+T\left(h_{k-1}\right)p_{\varphi_{k-1}}$ and $H_{x_{s,k}}=\left[\begin{array}{cc} A\left(h_{k-1}\right)H_{x_{s,k-1}} & T\left(h_{k-1}\right)H_{\varphi_{k-1}} \end{array}\right].$ The proof process is the same as Lemma 1 and the estimation of $x_{s,k}$, which can be described by

(30) $${\hat{x}}_{s,k}=\langle p_{{\hat{x}}_{s,k}},H_{{\hat{x}}_{s,k}}\rangle$$

where

$$\begin{array}{cc} p_{{\hat{x}}_{s,k}} & =\left(I_{n}-\tilde{\Lambda }\left(h_{k-1}\right)C\right)A\left(h_{k-1}\right)p_{{\hat{x}}_{s,k-1}}+\left(I_{n}-\tilde{\Lambda }\left(h_{k-1}\right)C\right)B\left(h_{k-1}\right)u_{k-1}\\ & \;+\left(I_{n}-\tilde{\Lambda }\left(h_{k-1}\right)C\right)T\left(h_{k-1}\right)p_{\varphi_{k-1}}+\tilde{\Lambda }\left(h_{k-1}\right)y_{s,k}-\tilde{\Lambda }\left(h_{k-1}\right)Fp_{v}\\ H_{{\hat{x}}_{s,k}} & =\left[\left(I-\tilde{\Lambda }\left(h_{k-1}\right)C\right)A\left(h_{k-1}\right)H_{{\hat{x}}_{s,k-1}}\;\left(I-\tilde{\Lambda }\left(h_{k-1}\right)C\right)T\left(h_{k-1}\right)H_{\varphi_{k-1}}\;-\tilde{\Lambda }\left(h_{k-1}\right)FH_{v}\right]. \end{array}$$

$\tilde{\Lambda }\left(h_{k-1}\right)\in R^{n\times p}$ is a new correction matrix in the following polytopic form: $\tilde{\Lambda }=\sum_{i=0}^{N}h_{i}\left(z\left(k\right)\right){\tilde{\Lambda }}_{i}.$

Now, the method of solving the correction matrix to obtain a system state estimation can be summarized as follows:

**Corollary 2.** 
*Consider the T-S fuzzy model (29) if there exists a symmetric positive-definite matrix ($\tilde{P}={\tilde{P}}^{T}\in R^{n\times n}$) and matrix (${\tilde{Y}}_{i}\in R^{n\times p}$) such that*

$$\begin{array}{cc} {\tilde{\Phi }}_{i,i} & >0,\;i=1,2,\ldots ,\lambda \\ \frac{2}{\lambda -1}{\tilde{\Phi }}_{i,i}+{\tilde{\Phi }}_{i,j}+{\tilde{\Phi }}_{j,i}\; & \geq 0,\;1\leq i<j\leq \lambda \end{array}$$

*and*

$$\left[\begin{array}{cc} I_{n} & *\\ \tilde{\gamma }\tilde{P} & \tilde{P} \end{array}\right]\leq 0$$

*with*

$$\begin{array}{c} {\tilde{\Phi }}_{i,j}=\left[\begin{array}{cccc} \alpha \tilde{P} & * & * & *\\ 0 & (1-\alpha )\beta & * & *\\ 0 & 0 & \left(1-\alpha )(1-\beta \right) & *\\ -\tilde{P}A_{i}+{\tilde{Y}}_{j}CA_{i} & -\tilde{P}D_{i}+{\tilde{Y}}_{j}CD_{i} & {\tilde{Y}}_{j}F & \tilde{P} \end{array}\right]. \end{array}$$

*Then, the worst-case bound for $\ell_{k}$ is obtained as $\ell_{k}\leq {\tilde{\gamma }}^{2}\left({\beta ∥\varphi ∥}_{\infty }^{2}+\left(1-\beta \right){∥v∥}_{\infty }^{2}\right)\;\mathrm{for}\;\tilde{\gamma }>0$, satisfying the optimization problem ($\min_{\tilde{P},{\tilde{Y}}_{i}}\tilde{\gamma }).$*

**Proof.**  The proof follows a procedure similar to those of Theorem 1 and Corollary 1, so it is omitted for brevity.

Next, following the same calculation process as in (30), we reconstruct a residual ($r_{s,k}$) as follows:

We denote ${\hat{x}}_{s,k}={\left[\begin{array}{ccc} {\hat{x}}_{1,s,k} & \cdots & {\hat{x}}_{n,s,k} \end{array}\right]}^{T},\;{\hat{p}}_{x_{s,k}}={\left[\begin{array}{ccc} {\hat{p}}_{1,x_{s,k}} & \cdots & {\hat{p}}_{n,x_{s,k}} \end{array}\right]}^{T}$ and ${\hat{H}}_{x_{s,k}}={\left[\begin{array}{ccc} {\hat{H}}_{1,x_{s,k}}^{T} & \cdots & {\hat{H}}_{n,x_{s,k}}^{T} \end{array}\right]}^{T};$ then, according to Property 3, we have ${\underline{x}}_{s,k}\leq x_{s,k}\leq {\bar{x}}_{s,k},$ where ${\bar{x}}_{s,k}={\left[\begin{array}{ccc} {\bar{x}}_{1,s,k} & \cdots & {\bar{x}}_{n,s,k} \end{array}\right]}^{T}$ and ${\underline{x}}_{s,k}={\left[\begin{array}{ccc} {\underline{x}}_{1,s,k} & \cdots & {\underline{x}}_{n,s,k} \end{array}\right]}^{T}$ with ${\underline{x}}_{j,s,k}={\hat{p}}_{j,x_{s,k}}-R_{d}\left({\hat{H}}_{j,x_{s,k}}\right)$ and ${\bar{x}}_{j,s,k}={\hat{p}}_{j,x_{s,k}}+R_{d}\left({\hat{H}}_{j,x_{s,k}}\right).$ Here, $R_{d}\left({\hat{H}}_{j,x_{s,k}}\right)$ returns the *j*th diagonal element of $R_{d}\left({\hat{H}}_{x_{s,k}}\right)$. Now, we define

(31) $$\begin{array}{cc} {\bar{y}}_{s,k} & =C^{+}{\bar{x}}_{s,k}-C^{-}{\underline{x}}_{s,k}+F^{+}\bar{v}-F^{-}\underline{v}\\ {\underline{y}}_{s,k} & =C^{+}{\underline{x}}_{s,k}-C^{-}{\bar{x}}_{s,k}+F^{+}\underline{v}-F^{-}\bar{v}, \end{array}$$

Then, we have ${\underline{y}}_{s,k}\leq y_{s,k}\leq {\bar{y}}_{s,k}\;\left(k\geq 0\right)$ when there are no faults in the system. With the output interval estimation obtained in (31), we develop a residual design method for detecting sensor and actuator faults. First, we define ${\bar{e}}_{y_{s}}=y_{s}-{\bar{y}}_{s}$ and ${\underline{e}}_{y_{s}}={\underline{y}}_{s}-y_{s}.$ Secondly, we define ${\bar{e}}_{y_{s},\max}=\max_{l\in \{1,\ldots ,p\}}\left({\bar{e}}_{y_{s},l}\right)$ and ${\underline{e}}_{y_{s},\max}=\max_{l\in \{1,\ldots ,p\}}\left({\underline{e}}_{y_{s},l}\right),$ where ${\bar{e}}_{y_{s},l}$ and ${\underline{e}}_{y_{s},l}$ are the *l*th elements of vectors ${\bar{e}}_{y_{s}}$ and ${\underline{e}}_{y_{s}}$, respectively. Thirdly, we define the residual used for fault-detection purposes as $r_{s,k}=\max\left({\bar{e}}_{y_{s},\max},{\underline{e}}_{y_{s},\max}\right)$.    □

Theoretically, based on the logical determination expressed as

(32) $$r_{s,k}=\left\{\begin{array}{cc} >r_{s,0}, & \mathrm{sensor}\;\mathrm{fault}\;\mathrm{occurrence}\\ <r_{s,0}, & \mathrm{no}\;\mathrm{sensor}\;\mathrm{fault}\;\mathrm{occurrence}, \end{array}\right.$$

it is possible to identify sensor faults in the system.

The complete algorithm procedure is presented below (Algorithm 1).
**Algorithm 1** Optimized Zonotopic Set-Membership Estimation, Fault Detection and Isolation for T-S Fuzzy Systems  1:Given initial state zonotope $X_{x_{0}}$, disturbance zonotope $X_{w}$ and measurement-noise zonotope $X_{v}$;  2:$X_{x_{k-1}}⇐X_{x_{0}}$;  3:Solve convex LMI optimization minγ under fuzzy stability and disturbance-attenuation constraints, obtain *P* and $Y_{i}$, then compute optimized correction matrices $\Lambda_{i}^{*}=P^{-1}Y_{i},\;i=1,\ldots ,N$;  4:**for** k:=1: end **do**  5:   Obtain premise variable $z_{k-1}$, input $u_{k-1}$ and measurement $y_{k}$;  6:   Compute fuzzy weights $h_{i}\left(z_{k-1}\right)$, construct time-varying correction matrix $\Lambda \left(h_{k-1}\right)=\sum_{i=1}^{N}h_{i}\Lambda_{i}^{*}$;  7:   Perform zonotopic prediction and measurement update to get the intersection zonotope:$$X_{{\hat{x}}_{k}}\left(\Lambda (h_{k-1})\right)=\langle p_{{\hat{x}}_{k}}\left(\Lambda (h_{k-1})\right),H_{{\hat{x}}_{k}}\left(\Lambda (h_{k-1})\right)\rangle ;$$  8:   Derive state interval bounds $x_{i,k}\in \left[{\underline{x}}_{i,k},{\bar{x}}_{i,k}\right]$$$\begin{array}{cc} {\bar{x}}_{i,k} & =p_{{\hat{x}}_{k},i}\left(\Lambda (h_{k-1})\right)+Rd{\left(H_{{\hat{x}}_{k}}\left(\Lambda (h_{k-1})\right)\right)}_{i},\\ {\underline{x}}_{i,k} & =p_{{\hat{x}}_{k},i}\left(\Lambda (h_{k-1})\right)-Rd{\left(H_{{\hat{x}}_{k}}\left(\Lambda (h_{k-1})\right)\right)}_{i}, \end{array}$$   where $p_{{\hat{x}}_{k},i}$ is the *i*-th entry of $p_{{\hat{x}}_{k}}$, and $Rd{\left(\cdot \right)}_{i}$ denotes the *i*-th diagonal element of radius-summation matrix $H_{{\hat{x}}_{k}}$.  9:   Substitute state bounds into Equation (26) to obtain output bounds ${\bar{y}}_{k},{\underline{y}}_{k}$;10:   Compute error vectors ${\bar{e}}_{y}=y_{k}-{\bar{y}}_{k}$, ${\underline{e}}_{y}={\underline{y}}_{k}-y_{k}$:$${\bar{e}}_{y,\max}=\max_{l\in \{1,\ldots ,n_{y}\}}\left({\bar{e}}_{y,l}\right),\;{\underline{e}}_{y,\max}=\max_{l\in \{1,\ldots ,n_{y}\}}\left({\underline{e}}_{y,l}\right),\;r_{d,k}=\max\left({\bar{e}}_{y,\max},{\underline{e}}_{y,\max}\right)$$11:   If $r_{d,k}>r_{0}$: fault is detected, activate two parallel zonotopic estimation branches to generate isolation residuals and identify fault types;12:   If $r_{d,k}\leq r_{0}$: fault-free, skip isolation procedure;13:   Update $X_{x_{k-1}}⇐X_{{\hat{x}}_{k}}$ for next iteration;14:**end for**

## 5. Simulation

All numerical simulations in this paper are implemented in MATLAB R2016b (MathWorks, Natick, MA, USA). This section considers a two-subsystem T-S fuzzy system, with its parameters expressed as

$$A_{1}=\left[\begin{array}{ccc} 0.5 & 0 & 0\\ 21 & 0.5 & 0\\ 1 & 2 & 0.5 \end{array}\right],\;A_{2}=\left[\begin{array}{ccc} 0.4 & 0 & 0\\ 1 & 0.5 & 0\\ 1 & 1 & 0.1 \end{array}\right],\;B_{1}=B_{2}=\left[\begin{array}{c} -0.7143\\ 0\\ 0.8 \end{array}\right],\;C=\left[\begin{array}{ccc} 1 & 0 & 0\\ 0 & -1 & 0 \end{array}\right].$$

$$D_{1}=D_{2}={\left[\begin{array}{ccc} 1 & 0 & 0 \end{array}\right]}^{T},\;F={\left[\begin{array}{cc} 0 & 1 \end{array}\right]}^{T},\;H={\left[\begin{array}{cc} 1 & 1 \end{array}\right]}^{T}\;\mathrm{and}\;w_{k}=0.7\sin\left(k\right),$$

$v_{k}=0.2\sin\left(k\right)$. The fuzzy weighting functions are specified as $h_{1}\left(x_{3}\right)=\frac{1-1/\left(1+\exp\left(-14\left(x_{3}-\pi /8\right)\right)\right)}{1+\exp\left(-14\left(x_{3}-\pi /8\right)\right)},\;$$h_{2}\left(x_{3}\right)=1-h_{1}\left(x_{3}\right).$ When conducting the simulation, we specify $u_{k}=3.3\sin\left(0.1k\right)$. Taking α=0.3 and β=0.6, the optimization problem (25) can be resolved with the aid of the LMI toolbox, and we obtain a minimum of γ=1.7245. Correspondingly, the positive-definite symmetric matrices (*P*) and the correction matrix ($\Lambda_{i}$(i=1,2)) are obtained as follows:

$$P=\left[\begin{array}{ccc} 1.2156 & 0.0823 & -0.2945\\ 0.0823 & 1.0187 & -0.1312\\ -0.2945 & -0.1312 & 1.1678 \end{array}\right],\;\Lambda_{1}=\left[\begin{array}{cc} 0.4012 & 0.1256\\ 0.0856 & 0.3512\\ 0.2615 & 0.1423 \end{array}\right],\;\Lambda_{2}=\left[\begin{array}{cc} 0.3895 & 0.1187\\ 0.0812 & 0.3365\\ 0.2503 & 0.1321 \end{array}\right].$$

**Figure 1:**
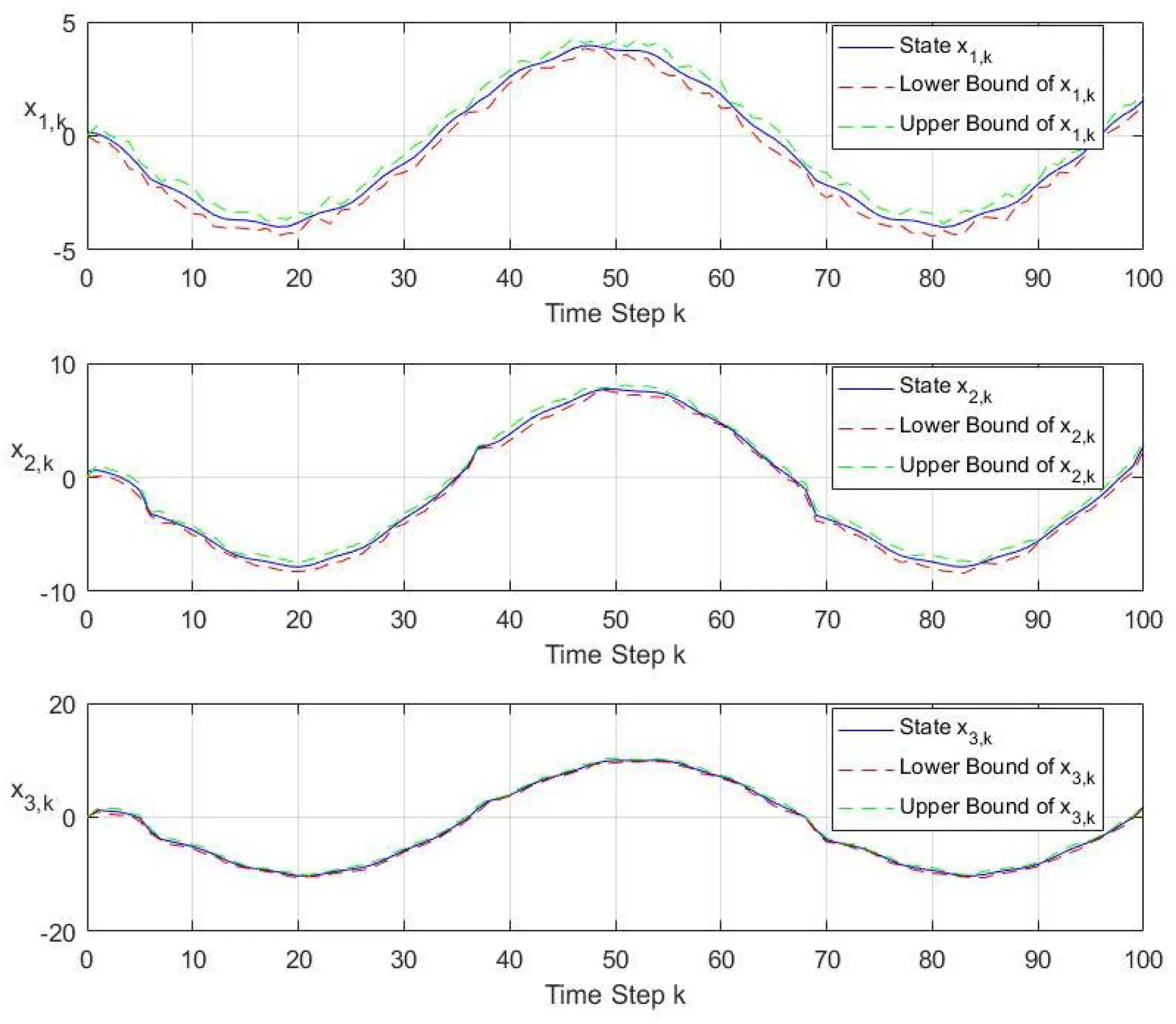
The interval state estimation result of *x*.

On the premise of $f_{a,k}\equiv 0$, as well as $f_{s,k}\equiv 0$, the state interval estimation obtained from zonotopic set-membership is shown in Figure 1. It can be seen that the state interval estimations are satisfactory.

In order to investigate the effectiveness of fault detection, we assume that $f_{s}\left(k\right)=\left\{\begin{array}{cc} 1, & 10\leq k\leq 30,\\ 0, & \mathrm{others} \end{array}\right.$ and $f_{a}\left(k\right)=\left\{\begin{array}{cc} 3, & 20\leq k\leq 50,\\ 0, & \mathrm{others} \end{array}\right..$

**Figure 2:**
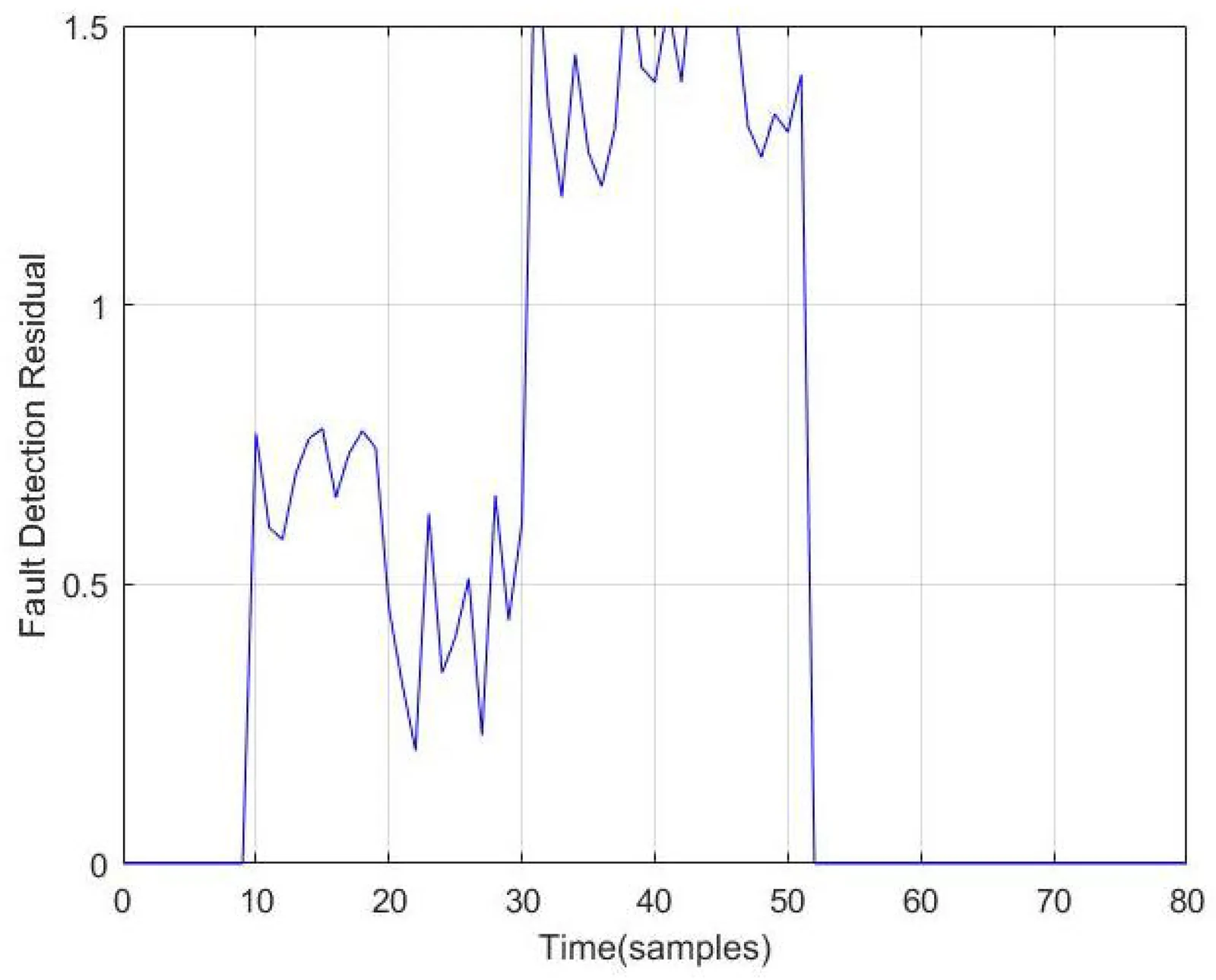
Fault detection when both sensor and actuator faults occur.

Figure 2 shows the residuals when sensor and actuator faults occur separately and simultaneously, with sensor faults active over [10,30] and actuator faults active over [20,50]. It can be seen that the proposed fault detection method hierarchically identifies fault severity by observing the residual ($r_{d,k}$) defined in (27). Mild sensor-induced residual fluctuations below the threshold are tolerated to avoid false alarms, while severe actuator faults driving residuals across the threshold are reliably detected. Here, the scalar fault-diagnosis threshold ($r_{0}$) is set to 0.8. This value reserves a proper safety margin above the maximum residual amplitude of the single sensor fault under fault-tolerant operation, following the threshold-tuning principle illustrated in Remark 2.

**Figure 3:**
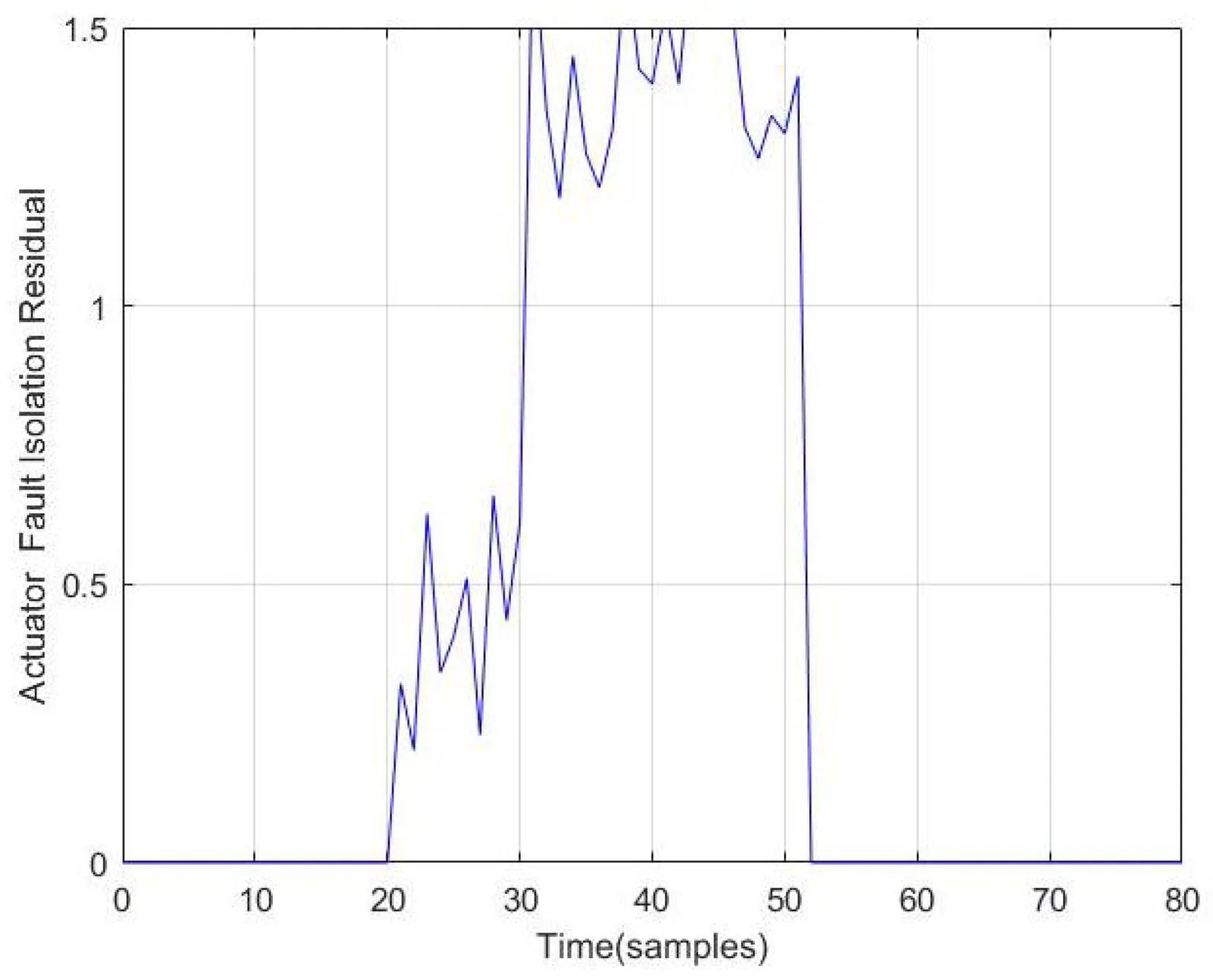
Simulation response of actuator fault isolation.

Then, we verify the Section 5 fault isolation method to distinguish sensor and actuator faults. The actuator fault isolation residual calculated by Equation (28) is shown in Figure 3. The residual rises evidently from 20 to 50 samples, accurately identifying the actuator fault interval and avoiding misjudgment of pure sensor faults.

For sensor faults, solving new LMI and optimization problems, a minimal value of $\tilde{\gamma }=1.8976$ is obtained. Correspondingly, the matrices ($\tilde{P}$) and the correction matrix (${\tilde{\Lambda }}_{i}\left(i=1,2\right)$) are determined as

$$\tilde{P}=\left[\begin{array}{ccc} 1.3256 & 0.0912 & -0.3125\\ 0.0912 & 1.0876 & -0.1423\\ -0.3125 & -0.1423 & 1.2345 \end{array}\right],\;{\tilde{\Lambda }}_{1}=\left[\begin{array}{cc} 0.4215 & 0.1423\\ 0.0987 & 0.3876\\ 0.2765 & 0.1523 \end{array}\right],\;{\tilde{\Lambda }}_{2}=\left[\begin{array}{cc} 0.4087 & 0.1356\\ 0.0945 & 0.3723\\ 0.2654 & 0.1412 \end{array}\right].$$

**Figure 4:**
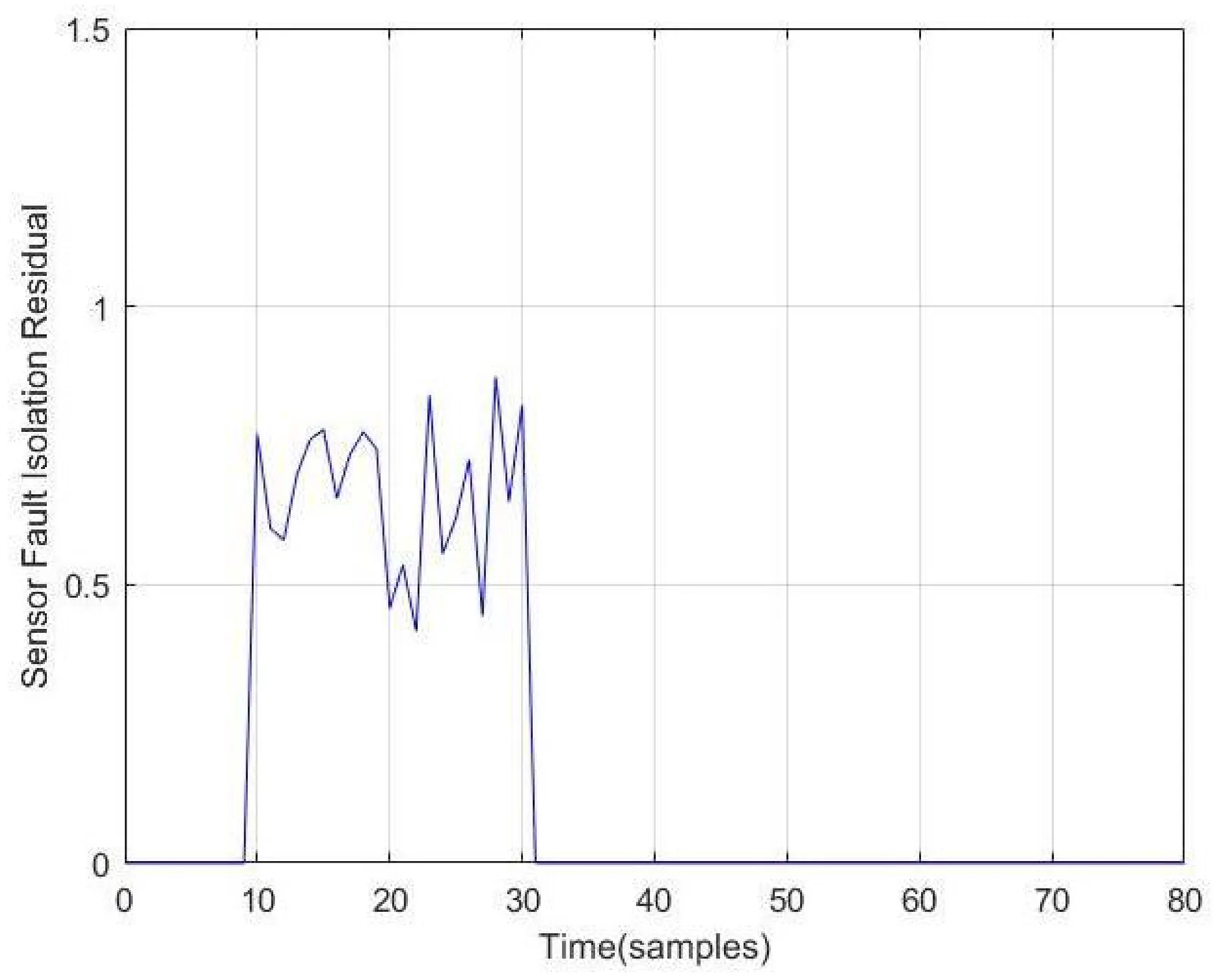
Simulation response of sensor fault isolation.

The sensor isolation residual ($r_{s,k}$) calculated by Equation (32) is plotted in Figure 4. The residual rises notably within 10–30 samples, accurately locating the sensor fault interval.

**Figure 5:**
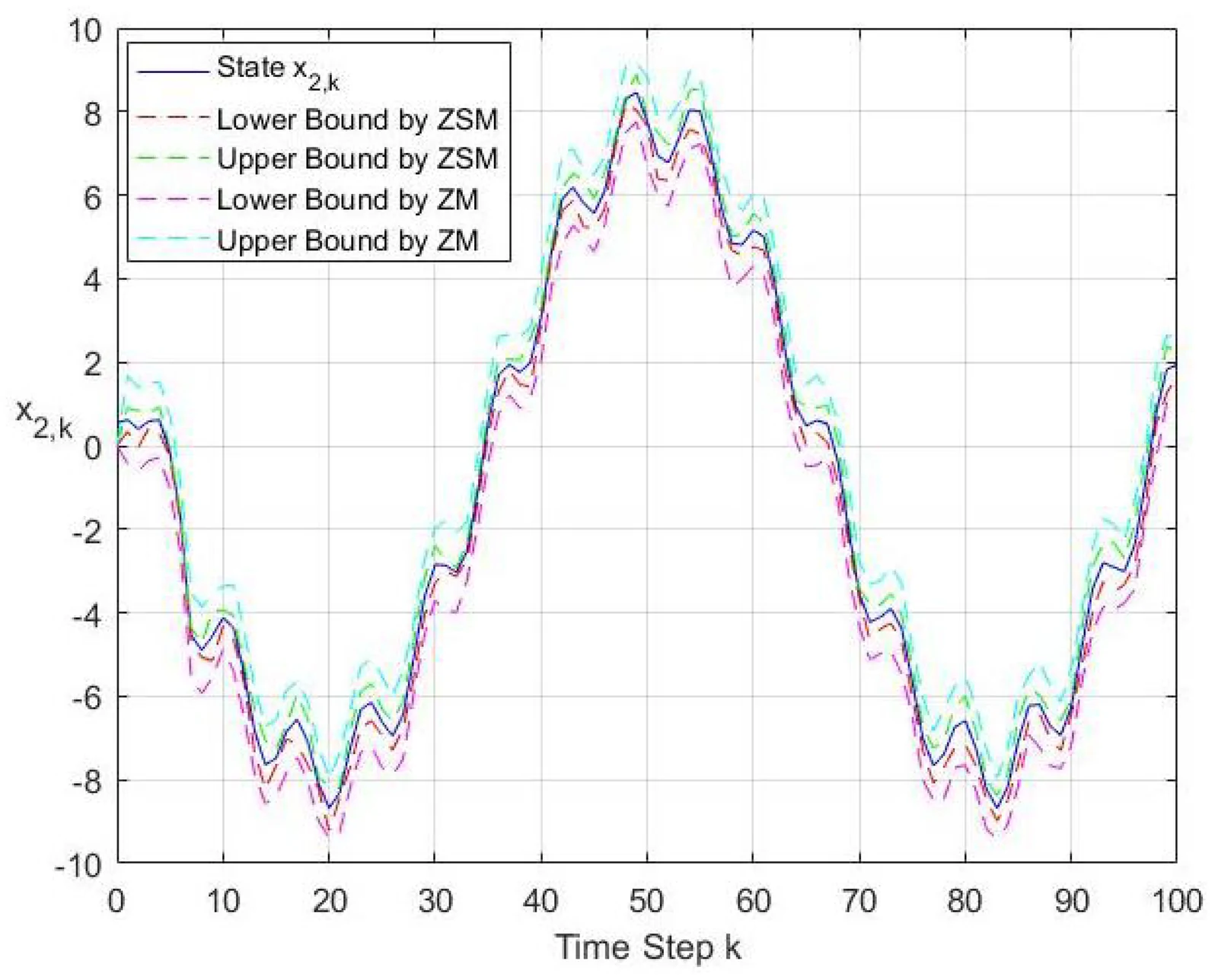
State interval estimation by ZSM and ZM.

Subsequently, a comparative study is conducted between the proposed zonotopic set-membership (ZSM) method and the traditional zonotope (ZM) method reported in Reference [26]. Figure 5 illustrates the interval estimation results of state $x_{2,k}$ obtained by the two approaches. It can be observed that both methods can effectively enclose the true trajectory of state $x_{2,k}$. Compared with the conventional ZM method, the ZSM method optimizes the zonotope via the correction matrix, which effectively narrows the width of the zonotopic interval and reduces the conservatism of estimation. As a result, the interval bounds fit the true state more closely, leading to superior accuracy of interval estimation.

**Figure 6:**
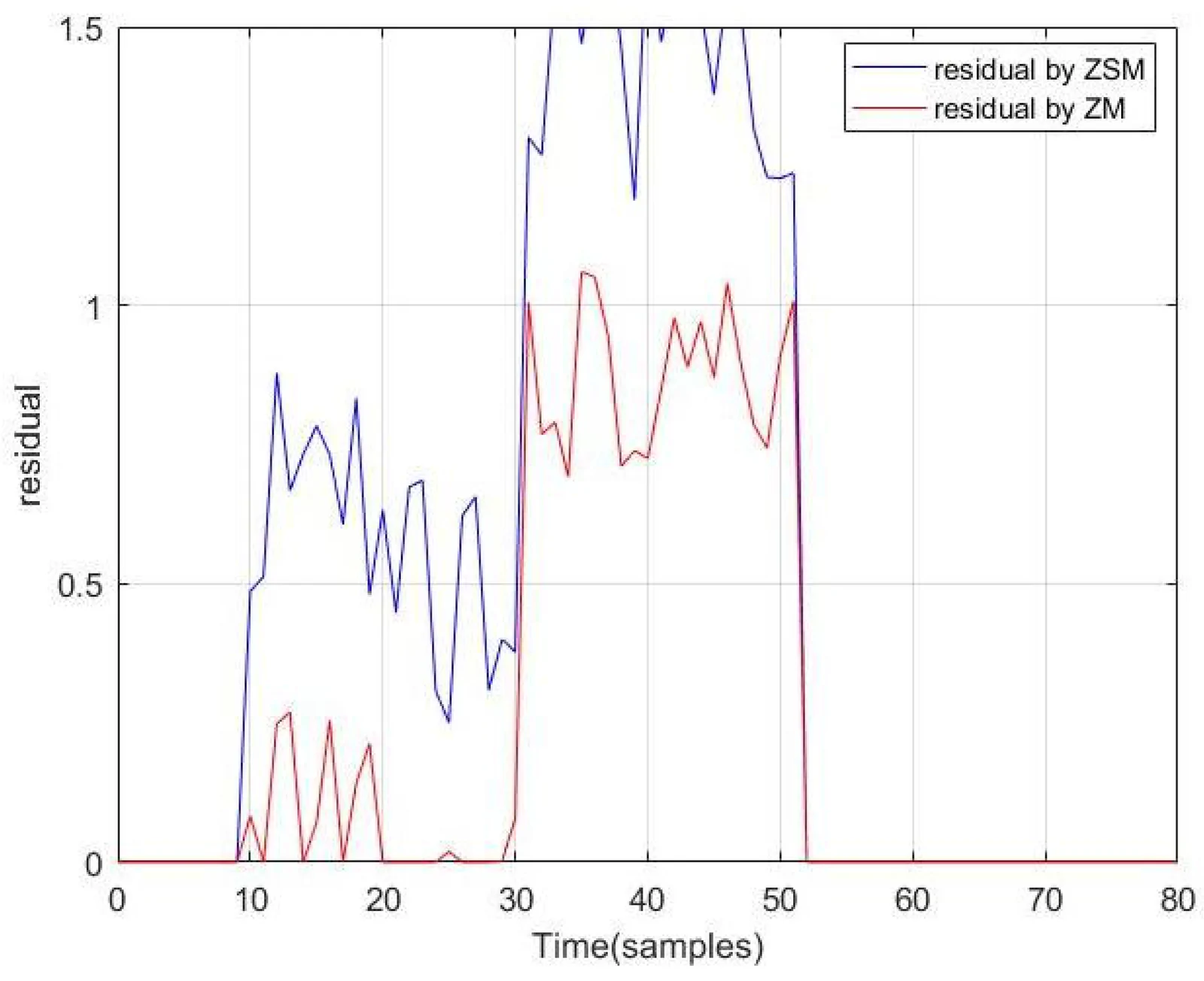
FD when ω(k)=0.1sin(k).

**Figure 7:**
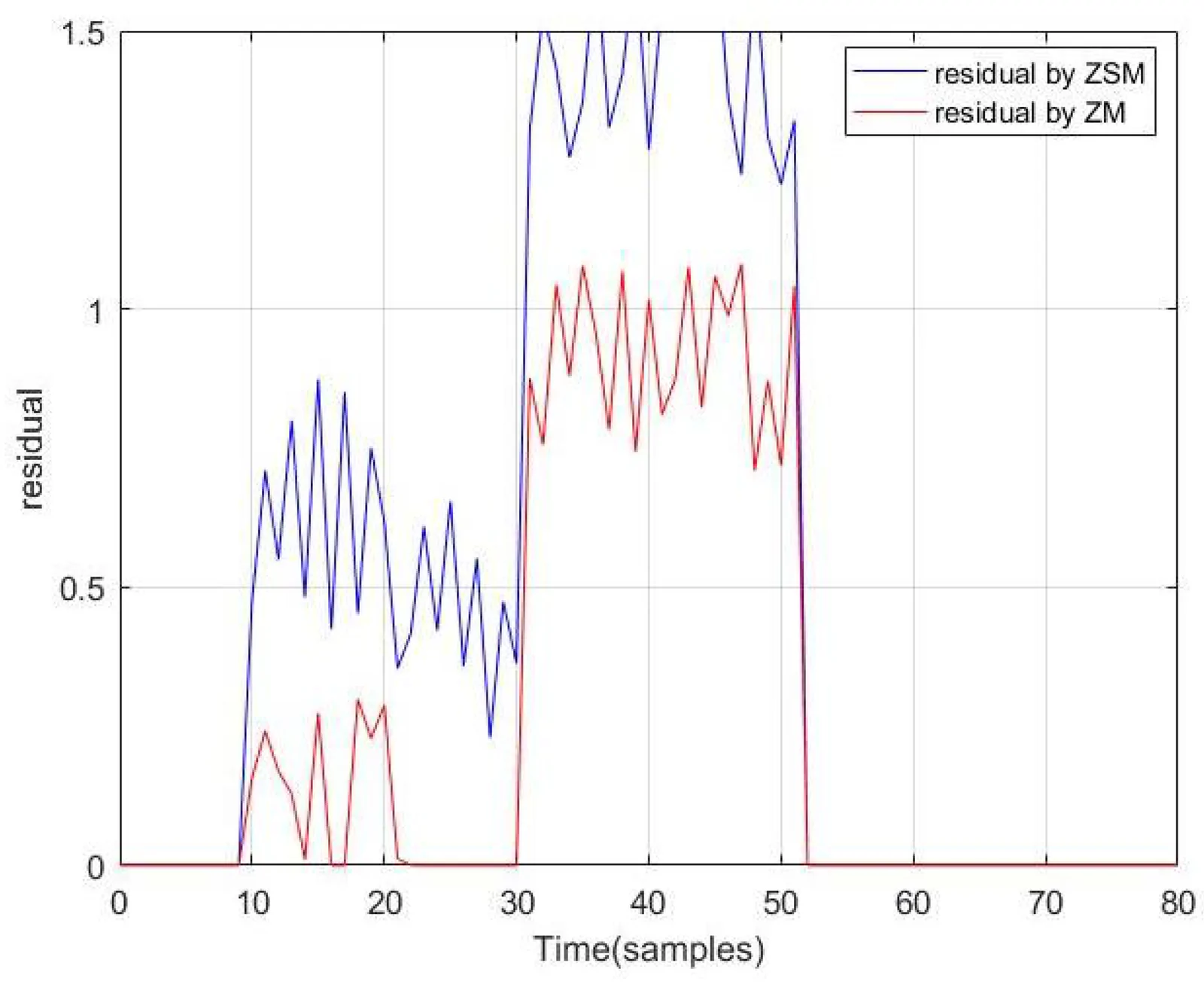
FD when ω(k)=0.4sin(k).

**Figure 8:**
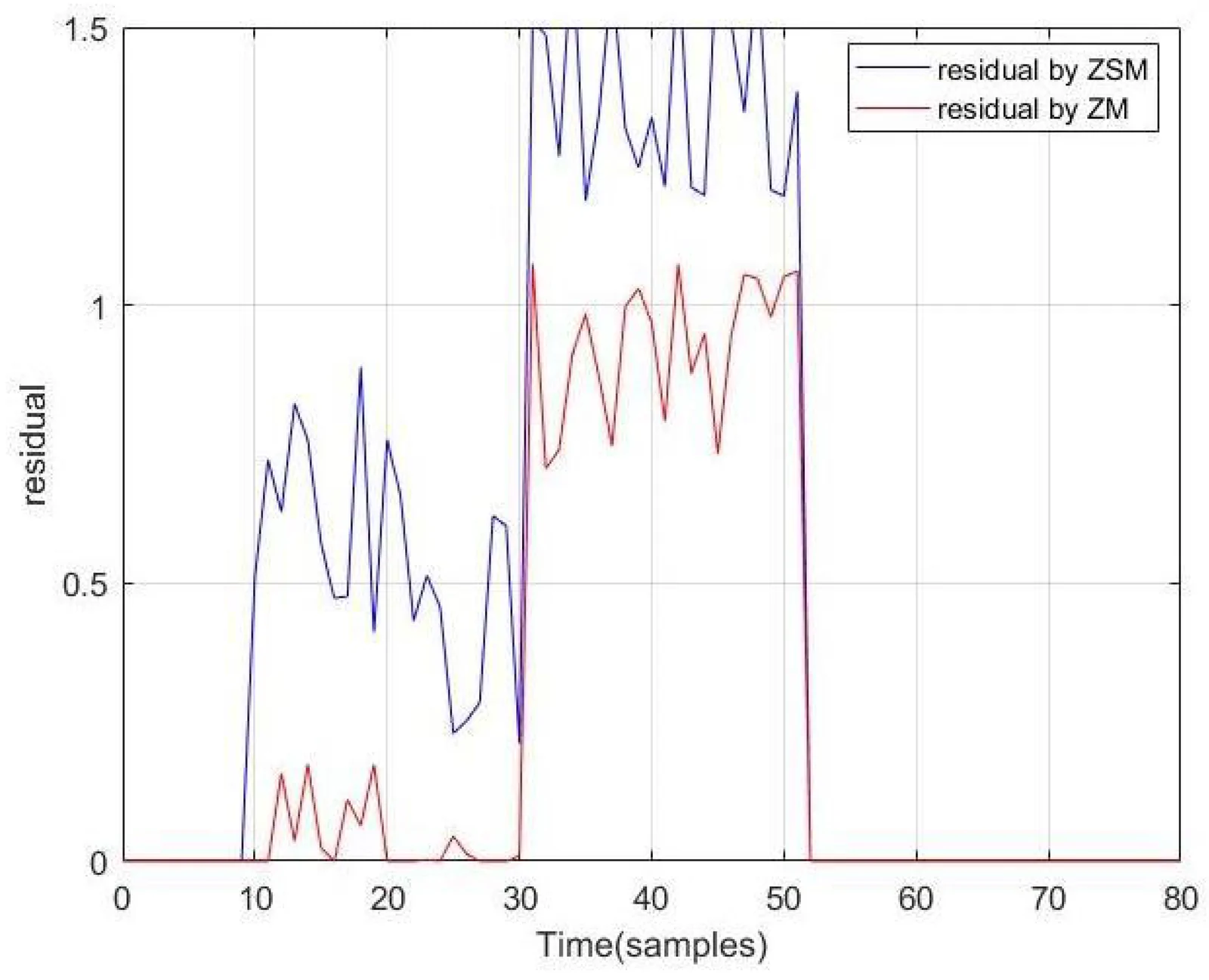
FD when ω(k)=0.7sin(k).

Figure 6, Figure 7 and Figure 8 compare the residual performance of ZSM and ZM under sinusoidal disturbances with amplitudes of 0.1, 0.4 and 0.7, with sensor faults in 10∼30 samples and actuator faults in 20∼50 samples. Under low disturbance, both methods detect actuator faults, and ZSM amplifies mild sensor-fault signatures while keeping residuals below $r_{0}=0.8$ without false alarms. With medium disturbance, ZM suffers from a reduced safety margin while ZSM maintains stable hierarchical detection. Under strong disturbance, weak fault features are blurred in ZM results, while ZSM distinguishes disturbances from faults: mild sensor anomalies will not trigger alarms, and severe actuator faults can be detected reliably. The superiority of ZSM over ZM becomes more evident as disturbance intensity rises, and ZSM achieves a good balance between false-alarm suppression and fault detection for practical applications.

Although the proposed ZSM method achieves higher fault detection sensitivity and stronger anti-disturbance robustness, this work still has limitations in experimental validation. All results were acquired via numerical simulations, without hardware-in-the-loop tests, physical experiments or benchmark dataset validation. This paper mainly provides qualitative mechanism comparisons; adequate quantitative comparative simulations across multiple operating conditions are insufficient, and supplementary systematic verification will be arranged in follow-up research.

## 6. Conclusions

For uncertain T-S fuzzy systems with both actuator and sensor faults, the problems of state interval estimation based on zonotopic set-membership approaches were investigated herein. On this basis, a fault detection and isolation (FDI) strategy was developed, relying on optimized set-membership construction. To achieve tighter state estimation bounds, a correction matrix was embedded into the zonotope update framework, where the $L_{\infty }$ performance index is adopted to constrain the minimum bounding radius and the desired correction matrix is obtained by solving linear matrix inequalities. Based on the optimized set-membership residuals, effective fault detection and isolation were realized. Nevertheless, the proposed approach has certain inherent limitations. It can only distinguish actuator faults from sensor faults, without locating specific faulty components. The numerical simulation simplifies practical industrial uncertainties, and the engineering adaptability remains to be further validated. Next, we will further refine the fault isolation algorithm to realize precise localization of faulty actuators and sensors. Meanwhile, we will integrate theoretical research with practical engineering applications to tackle fault-diagnosis difficulties in actual operation scenarios of industrial robots.

## Figures and Tables

**Table 1:** Performance comparison of different fault-diagnosis methods.

Index	Proposed Method(Zonotope Set-Membership Estimation)	Ref. [10](Adaptive Sliding-Mode Observer)	Ref. [28](Zonotope Mathod Based on Interval Observer)	Ref. [30](Unknown-Input Observer with Zonotope Analysis)
Interval Estimation	Time-varying interval estimation with low conservatism	Only point estimation; no interval output	Only residual interval; lacking complete state interval	Basic interval estimation with high conservatism
Fault Diagnosis	Dynamic threshold; robust to time-varying disturbances	Fixed threshold; weak anti-noise ability; prone to misjudgment	Strong disturbance decoupling; only applicable to interval type-2 fuzzy systems	Detection accuracy limited by observer performance
Fault Isolation	Effective isolation for coupled actuator and sensor faults	Available isolation but sensitive to model uncertainty	Only fault detection, without isolation capability	Basic isolation; poor adaptability to coupled faults
Computational Cost	Moderate and controllable computation via dual-branch LMI optimization	Low computational complexity with simple structure	High overhead caused by complicated LMI constraints	Heavy computational burden with potential numerical chattering

## Data Availability

The original contributions presented in this study are included in the article. Further inquiries can be directed to the corresponding author.
